# Systems Engineering Approach to Modeling and Analysis of Chronic Obstructive Pulmonary Disease

**DOI:** 10.1021/acsomega.3c00854

**Published:** 2023-05-26

**Authors:** Varghese Kurian, Navid Ghadipasha, Michelle Gee, Anais Chalant, Teresa Hamill, Alphonse Okossi, Lucy Chen, Bin Yu, Babatunde A. Ogunnaike, Antony N. Beris

**Affiliations:** † Department of Chemical and Biomolecular Engineering, University of Delaware, Newark, Delaware 19716, United States; ‡ Daniel Baugh Institute of Functional Genomics/Computational Biology, Department of Pathology and Genomic Medicine, Thomas Jefferson University, Philadelphia, Pennsylvania 19107, United States; § American Air Liquide Inc., Innovation Campus Delaware, Newark, Delaware 19702, United States

## Abstract

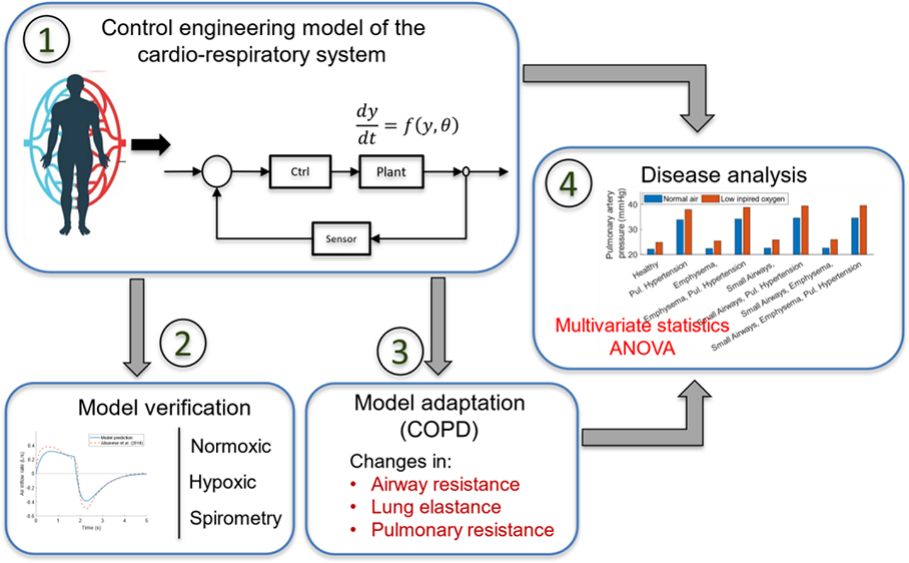

Chronic obstructive pulmonary disease (COPD) is a progressive lung disease characterized by airflow limitation. This study develops a systems engineering framework for representing important mechanistic details of COPD in a model of the cardiorespiratory system. In this model, we present the cardiorespiratory system as an integrated biological control system responsible for regulating breathing. Four engineering control system components are considered: sensor, controller, actuator, and the process itself. Knowledge of human anatomy and physiology is used to develop appropriate mechanistic mathematical models for each component. Following a systematic analysis of the computational model, we identify three physiological parameters associated with reproducing clinical manifestations of COPD: changes in the forced expiratory volume, lung volumes, and pulmonary hypertension. We quantify the changes in these parameters (airway resistance, lung elastance, and pulmonary resistance) as the ones that result in a systemic response that is diagnostic of COPD. A multivariate analysis of the simulation results reveals that the changes in airway resistance have a broad impact on the human cardiorespiratory system and that the pulmonary circuit is stressed beyond normal under hypoxic environments in most COPD patients.

## 1 Introduction

“Chronic obstructive pulmonary disease (COPD) is a common, preventable, and treatable disease that is characterized by persistent respiratory symptoms and airflow limitation”.^1^ The airflow limitation that is due to either or a combination of bronchial and alveolar abnormalities is usually caused by significant exposure to noxious particles and gases such as cigarette smoke.^2^ Chronic cough, excessive sputum production, and dyspnea are the most prevalent symptoms in COPD patients.^3^ Both pharmacological (bronchodilators, anti-inflammatory therapy) and nonpharmacological (exercise training, oxygen therapy, education) interventions are available for management of the condition and slowing the progress of the disease.^1^ These are, however, not adequate, and in general, patients experience a poorer quality of life and an increased risk of death.^4^ Recent estimates of COPD indicate a global prevalence of about 40 crores (0.4 billion, 11.7% of population with age ≥30) and annual deaths of over 30 lakhs (3 million).^5,6^ Despite being a major public health challenge, there exist several impediments in the recognition, assessment, and management of the condition.^7,8^

There are three common manifestations of COPD: small airways disease, emphysema, and pulmonary hypertension (PH).^1,9^ Patients with small airways disease are faced with difficulty in exhaling, which progressively traps gas in the lungs causing dynamic hyperinflation. This increase in lung volume is often associated with increased dyspnea and exercise limitation.^1^ Emphysema is an abnormal permanent enlargement of air sacs distal to the terminal bronchioles in the lungs. This enlargement can destroy the airspace walls, without obvious pulmonary fibrosis (i.e., there is no fibrosis visible to the naked eye).^10^ Additionally, in COPD patients, significant abnormalities are observed in the microvascular blood flow,^9^ which cause an increased pulmonary blood pressure resulting in PH. The stress associated with PH can lead to right ventricular hypertrophy (increase in the muscle mass of right ventricle) and eventually, cardiac failure. The relative contributions of these disease manifestations vary from person to person and may also evolve at different rates over time. This heterogeneity in disease trajectories along with the variations in response to therapy has promoted the emergence of personalized medicine^11,12^ as an effective tool for the management of COPD.^13^ However, at present, there are several impediments to this including the limitations in our understanding of the disease pathophysiology and the lack of biomarkers.^13^

Although there is no single definitive test for the diagnosis of COPD, spirometry is a common method which is used to measure airflow obstruction in COPD patients. Despite its high sensitivity in diagnosing COPD, spirometry cannot be reliably used as the only diagnostic test because of its weak specificity.^14^ For this reason, other parameters such as symptoms and risk factors are also considered in conjunction with spirometric data. Efforts are also being made to evaluate the usefulness of radiographic measurements in the diagnosis of COPD, and to identify any biomarkers that are predictive/diagnostic of COPD.^15^

*Mathematical models* are useful in analyzing the information contained in the physiological measurements and making appropriate inferences on the disease state of the patients. In this way, the models could assist in the personalization of COPD management strategies and help realize the clinical and economic benefits of *remote patient monitoring* – the collection and secure transmission of health data from individuals in one location to healthcare providers in a different location for assessment and recommendations.^16^ Though there have been a few recent efforts toward developing models of the cardiorespiratory system,^17−21^ these have not been adapted to capture the response of COPD patients. For example, the model by Gutta et al.^21,22^ uses reduced equations to represent certain aspects of the respiratory mechanics, the details of which are critical for representing the adaptations in COPD patients (but not for studying sleep apnea, the original application of the model). A more detailed model by Albanese et al.^17^ assumes that the volume of air inhaled and exhaled in different respiratory cycles are the same, making it difficult to model the dynamics of air entrapment, a common symptom in COPD. The reduced order mechanistic model of the lung developed by Abbasi and Bozorgmehry Boozarjomehry^23^ does not include the control and circulatory systems. On the other hand, the available models of COPD are either overly simplified, often ignoring important physical phenomena^24^ or excessively detailed, making it almost impossible to use them at a systemic scale.^25^ To the best of our knowledge, there exists no mathematical model of the human cardiorespiratory system that can provide insights into and make predictions of the systemic response of COPD patients to external stimuli.

We seek to bridge some of the gaps mentioned above by developing a physiology-based model of appropriate components of the cardiorespiratory system, using principles of systems and control engineering. Specifically, we propose modeling the occurrence of COPD from a control engineering perspective, whereby the cardiorespiratory system is represented as control system components whose physiological functions will be represented by appropriate mathematical equations (Section 2). The performance of the proposed cardiorespiratory model is tested through some simulation case studies involving healthy individuals and a comparison to experimental data from the literature (Section 3). On the premise that the disease state emerges as a malfunction of one of these biological functions,^26^ a list of model parameters associated with the manifestations of COPD is identified and the changes in value are quantified (Section 4). In the discussion section, Section 5, a systematic multivariable analysis of the effect of changes in the parameters on cardiorespiratory variables is investigated. Finally, in Section 6, we present our conclusions.

## 2 Control Engineering Model of the Cardiorespiratory System

Understanding the dynamics of the respiratory and cardiovascular systems, as well as their interactions, is essential to understand the underlying mechanisms of COPD. There is an increasing body of evidence that the inflammation associated with COPD is not limited to the lungs but can also affect nonpulmonary organs, in particular, the heart.^27,28^ Therefore, the model should provide adequate physiological insights into both cardiovascular and respiratory systems.

**Figure 1:**
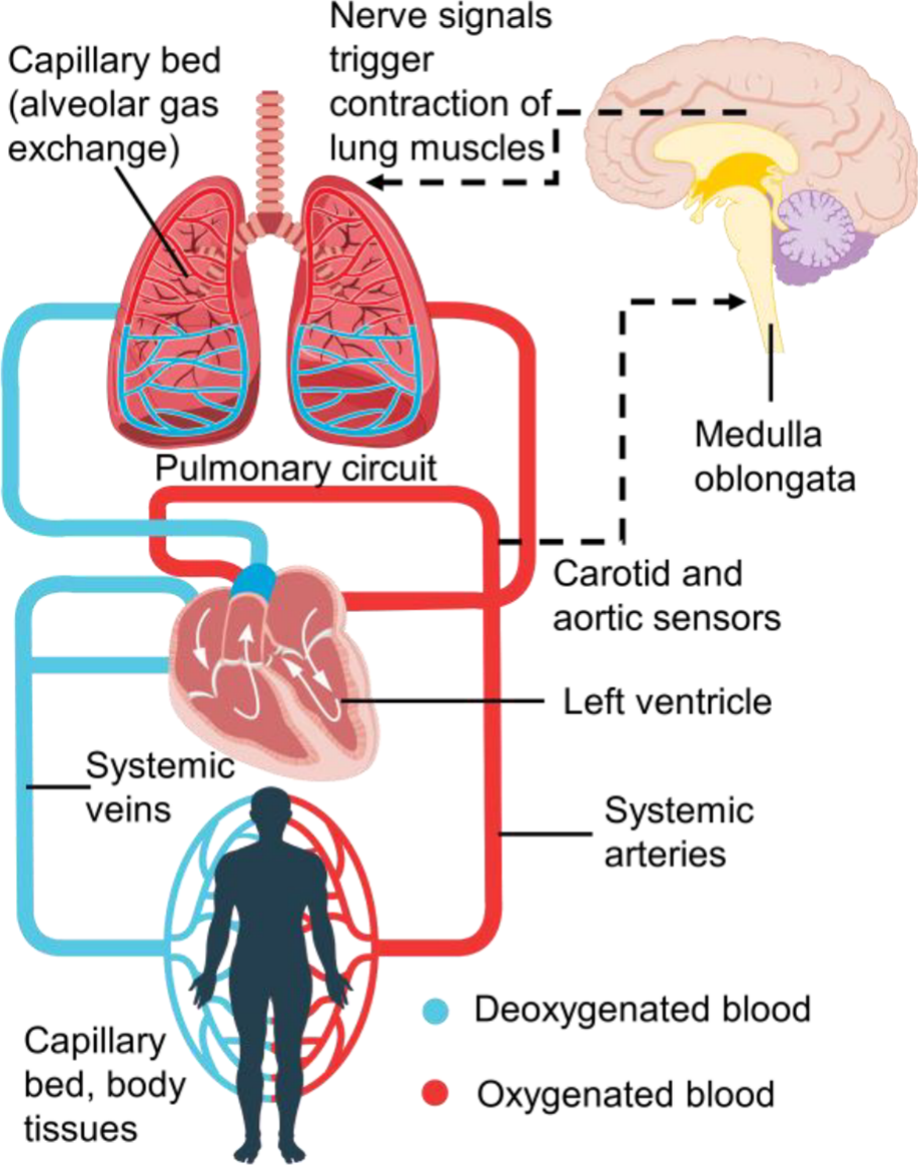
Schematic of the physiological components and mechanisms involved in breathing regulation and blood circulation. Image of cardiorespiratory system: Adapted with permission from istockphoto.com. Copyright 2022, istockphoto.com/colematt. Image of brain: Adapted with permission from Cancer Research UK; obtained via Wikimedia Commons. Copyright 2014, Cancer Research UK.

Figure 1 shows a schematic of respiration and blood circulation in the body. As the blood moves from the lungs to the heart, then to the systemic networks, the oxygen concentration decreases, and the CO_2_ concentration increases. Those are rectified in the lungs through the breathing process. Conceptually, the regulation of breathing is affected by an inherent biological control system consisting of complex interactions between the cardiorespiratory centers in the brain.^29−32^ The harmonious interactions of these individual components generate the breathing rhythm and regulate the levels of oxygen and carbon dioxide in the body.^33^ When the oxygenated blood flows through the systemic arteries, blood gas levels (concentration of oxygen and carbon dioxide) are measured by the respiratory sensors on the arteries. Respiratory sensors are chemoreceptors, which can detect changes in the chemical concentrations of blood gas levels.^34^ The chemoreceptors send the measured values of blood gas levels as an electrical signal to the respiratory control center, which is located in the medulla oblongata in the brainstem. Based on the measured values, the controller generates another electrical signal which determines the contraction of the lung muscles. The lung muscles apply the pleural pressure, adjusting the breathing frequency and depth.

**Figure 2:**
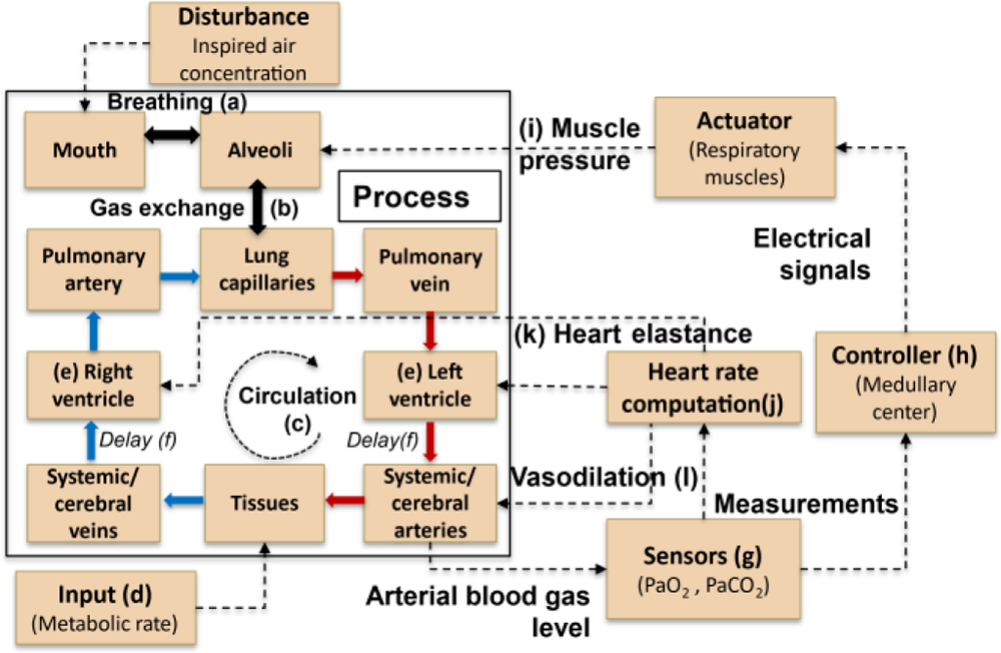
Control engineering block diagram representation of the cardiorespiratory system. Dashed lines describe the flow of information while the solid black, blue, and red lines are the flow of gas, deoxygenated, and oxygenated blood, respectively.

Figure 2 shows a control engineering representation of the above-mentioned components in the form of modules/blocks that perform the physiological functions of the sensor, controller, actuator, and process itself. This representation is particularly useful for the clarity with which it shows how each subsystem performs its function and how the various subsystems are connected such that the response (“output”) of one subsystem provides the stimulus (“input”) to another in the feedback loop. Specifically, the representation shows: (1) the *process:* how the “controlled variables” (or CVs, the physiological variables desired to be controlled), blood partial pressure of oxygen and carbon dioxide in the blood, are influenced by changes in the “manipulated variable” (MV), pleural pressure; (2) the *actuator*: how the MV—pleural pressure—in turn is determined by the “control action”—the electrical signal from the neural controller determines the contraction of the lung muscles; (3) the *controller*: how the control action signal to the lungs is determined in the control center in the brain, in response to the difference between the actual measured values of each of the CVs and their corresponding desired values; and (4) the *sensor*: how the measurements of the CVs are determined in the peripheral and central chemoreceptors, in response to changes in these CVs, and thereby closing the loop.

The mathematical expressions representing the mechanisms involved in the physiological function of each component module are derived from fundamental principles of material and momentum balance. The resulting differential-algebraic equations (summarized in Table 1, full set of equations available in Supporting Information, SI) show the output (response) of each block as a function of the corresponding input (stimulus) explicitly. In addition to the noted process variables, the model also consists of fixed parameters associated with the physiological characteristics of each component. The parameter values used in this work (listed in the SI) are either taken directly from the literature or estimated using the input–output data of the corresponding physiological process. In reality, the specific values taken by these parameters depend on the patient in question and contribute to the model prediction of the response to any specified stimulus. Hence, the model response reported in this work is representative of the population-average rather than a specific individual (see ref (35) for a technique to generate a patient ensemble from the existing parameters). It may be noted that, in the future, real patient data can be used to create personalized models by first identifying parameters that are *sensitive*(36,37) to the system outputs such as heart and respiratory rates, blood pressures, and spirometry results and then fitting those parameters to patient data.^37^

**Table 1:** Qualitative Description of the Models Used for Representing Key Physiological Processes and the Main References^a^

	physiological process	model equation	eq no.	main source
a	ventilation	pressure driven flow	S.1–S.4	(38,39,23)
b	gas exchange in lungs	diffusive transport	S.5–S.15	(38−40)
c	blood circulation	R-C circuit	S.16–S.19	(40)
d	metabolism	O_2_ and CO_2_ mass balance	S.20 and S.21	(40)
e	ventricle mixing	CSTR	S.22	(41)
f	circulatory delay	flow delay	S.23	(42)
g	sensors	noiseless linear relation	S.24–S.26	(39,43,44)
h	respiratory control	Ben-Tal method	S.27–S.36	(39,45)
i	displacement of lung muscles	linear ODE	S.37 and S.38	(38,39)
j	heart rate	polynomial	S.39 and S.40	(46)
k	elastance of heart muscles	trigonometric functions	S.41	(40)
l	vasodilation	sigmoid of heart rate	S.42	(47,48)

a The letters in the first column correspond to the symbols used in Figure 2. The equation numbers in the fourth column refer to the SI.

The integrated cardiorespiratory model is a system of differential-algebraic equations with a few algebraic constraints. The model was developed using Simulink/MATLAB R2020b and solved using the MATLAB function ode23 that uses the Bockagi–Shampine method with a relative tolerance of 10^–6^.^49^

## 3 Simulation Results: Healthy Individuals

### 3.1 Stationary Simulation Results under Standard Conditions

**Figure 3:**
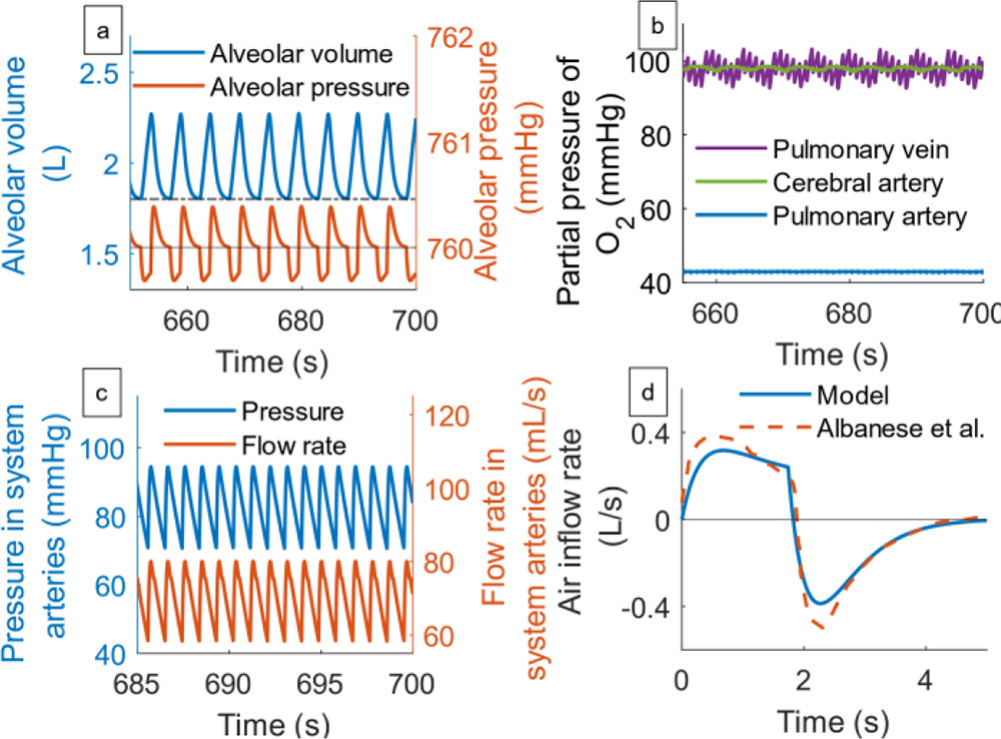
Dynamics of (a) alveolar volume (blue) and pressure (red), (b) concentration of oxygen in blood – pulmonary vein (purple), cerebral artery (green) and pulmonary artery (blue) (c) arterial pressure (blue), and blood flow (red), at TPSS. (d) Comparison of air inflow rate in normal breathing of the current model (solid blue) with the Albanese et al.^17^ model (dashed red). Air inflow rate redrawn with permission from the American Physiological Society. Copyright 2016, The American Physiological Society.

The first step toward verification of the model involved comparing the key process variables with their corresponding standard values for healthy individuals. For this, the mathematical model was simulated with the initial conditions given in the Supporting Information. As it has been reported earlier in the literature, the respiratory model requires several hundreds of seconds to converge to a *time periodic stationary state (TPSS)*.^40^ In our case, we chose a settling time of 500 s, as it was identified to be the most appropriate on analysis of the simulation data. In particular, after 500 s, no unsettled slow dynamics were observed. Hence, the model was simulated for a total of 700 s, and out of this, the state of the system in final 200 s were used for comparison with standard values. The TPSS behavior of a few respiratory and circulatory variables is shown in Figure 3.

At TPSS, the heart rate was 64 beats per minute and the respiratory rate was 11.7 breaths per minute representing normal values for a healthy individual. Figure 3a shows the variations in alveolar volume and the alveolar pressures. The alveolar volume and alveolar pressure form periodic functions that repeat with each cycle of inhalation and exhalation. The alveolar volume increases when the alveolar pressure is lower than 760 mmHg and decreases if it is higher. Figure 3b shows the oxygen concentrations within the circulatory system. There is a notable difference in the oxygen levels between the pulmonary arteries and veins due to the exchange of gases happening at the lungs and the body tissues. The periodicity due to both heart and respiratory rates is evident in the arterial gas concentrations. The dynamics due to the respiratory cycles are most prominent in the pulmonary veins and these almost vanish by the time the blood reaches pulmonary arteries due to the mixing happening in the ventricles. The faster dynamics due to the cyclic beating of the heart are evident in the oxygen concentrations of pulmonary veins. Figure 3c shows the profiles of blood pressure and flow rates in systemic arteries which are periodic with the heart rate. The blood pressure varies between 70 and 95 mmHg which is close to the normal range of 80–120 mmHg.^40,45^ The mean arterial flow rate of 69 mL/s is close to the average value of 67.6 mL/s mentioned in^40^ (pulmonary flow would be higher as it includes cerebral flow). Figure 3d shows the model predictions (solid line) for the inflow rate of air in one respiratory cycle. The expiratory flow rate asymptotically approaching zero toward the end of the respiratory cycle is representative of the passive exhalation in humans. On the other hand, the transition from inhalation to exhalation is more rapid due to the abrupt relaxation of the muscles. The profile is also in agreement with the model by Albanese et al.^17^ shown by the dashed line.

### 3.2 Decreased Level of Inspired O_2_ Concentration

In the next step, we tested the performance of the proposed cardiorespiratory model under varying environmental conditions, similar to the experiments performed by Dripps and Comroe^50^ It is important to emphasize that we have not tried to fit any parameters in the model to mimic the specific experiment.

**Figure 4:**
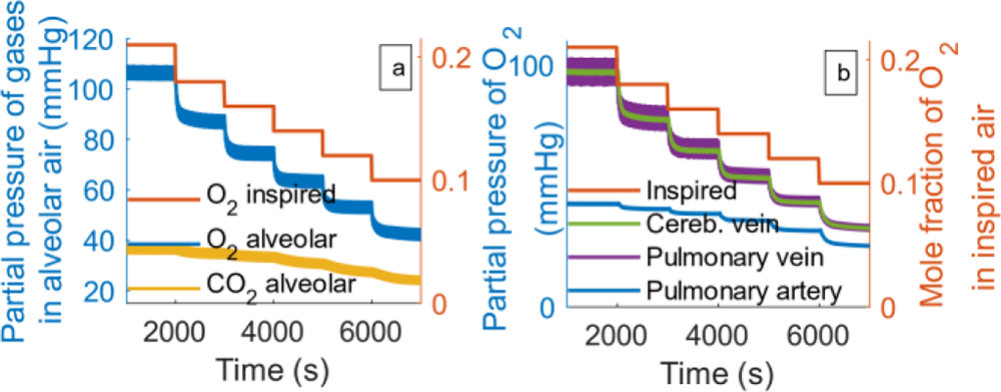
Changes with respect to time of (a) alveolar gas concentrations and (b) partial pressure of oxygen in blood. The right axis (orange line) shows the variations of the imposed inspired oxygen concentration.

**Figure 5:**
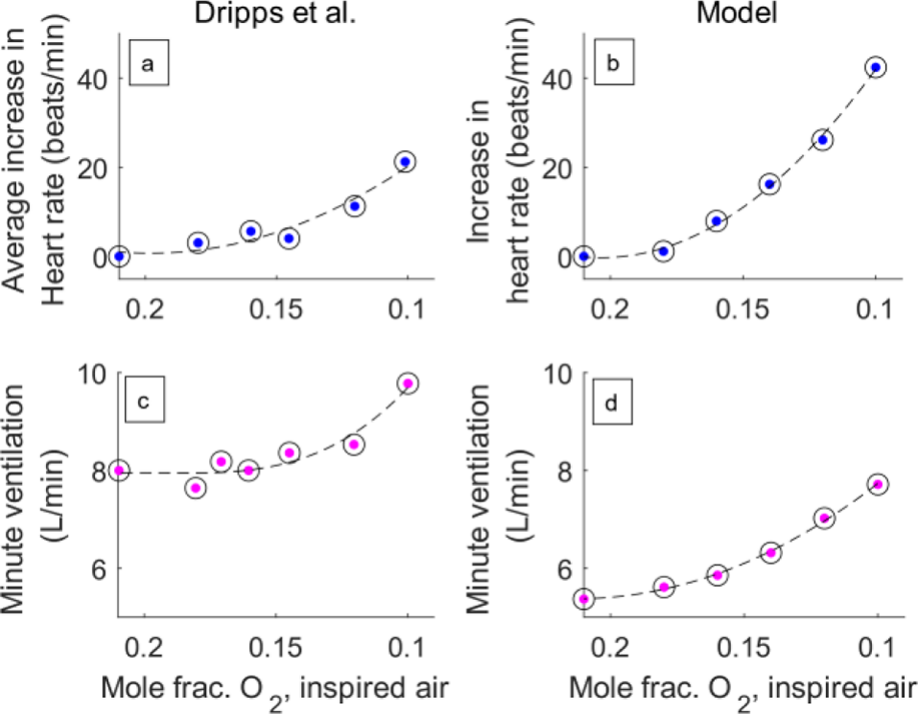
Variations with respect to the imposed inspired oxygen concentration in heart rate and minute ventilation measured by Dripps and Comroe.^50^ (a, c) and as obtained from simulations (b, d). Reference profiles redrawn with permission from the American Physiological Society. Copyright 1947, The American Physiological Society.

Dripps and Comroe,^50^ determined the response of the human respiratory and circulatory systems to anoxemia—deficiency of oxygen in the arterial blood—by exposing their subjects to atmospheres with low oxygen concentrations such as commonly observed in airplanes or at high altitudes. In this experiment, the oxygen concentration in inspired air was dropped from normal levels to 10% in a stepwise fashion with respect to time as shown in Figure 4. In the figure, we can see that gas concentrations of not just O_2_, but also CO_2_ in the alveoli and the blood progressively decreased. The CO_2_ behavior is due to the increased ventilation (shown later in Figure 5) that promotes the easy removal of carbon dioxide. Note that the model includes transport delays which cause the dynamics observed in the pulmonary arteries to fall behind those of the pulmonary veins, which however, is not very evident in Figure 4 due to the scale of the plot.

Figure 5b,d shows the TPSS values of changes in heart rate and minute ventilation given by the model. These were obtained by calculating the average of the heart and respiratory rates in the final 200 s at each level of inspired oxygen concentration. Both heart and respiratory rates increased with decreased oxygen levels in inspired air and their gradients were also higher at lower oxygen levels, as observed by Dripps and Comroe^50^ (Figure 5a,c).

### 3.3 Replicating a Spirometry Test

**Figure 6:**
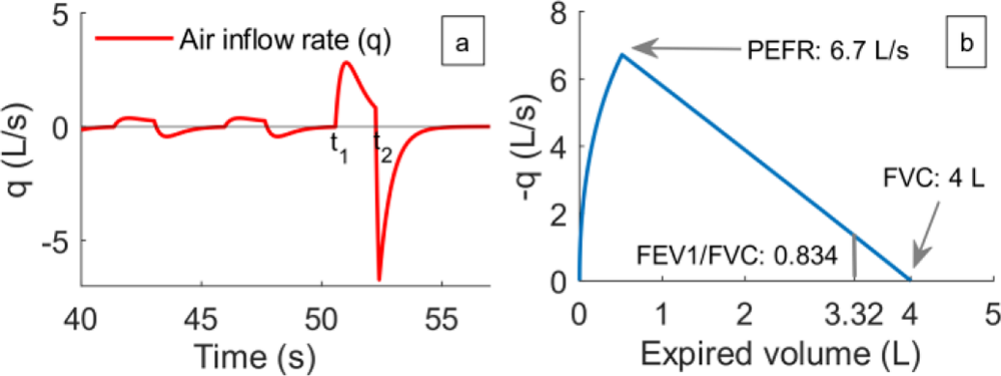
(a) Air inflow rate with respect to time during a spirometry. (b) Spirogram showing the flow rate with respect to expired volume. The expired volume is obtained as the integral of −*q* starting at time *t*_2_.

In COPD patients, the obstruction to airflow in the lungs is usually diagnosed using a spirometry test. During this test, the patients are asked to take a deep inhalation and then exhale the air as quickly as they can into a spirometer. The spirometer measures the air capacity in the lungs and how fast the person expires the air, and calculates important respiratory variables such as forced vital capacity (FVC), FEV1 ratio (the ratio of the volume of air expired out in 1 s during a forced expiration to that of the total volume of air expired out: ), and peak expiratory flow rate (PEFR). A significant amount of work has been done on the correlation between these variables and the severity of COPD^.^^51,52^ In the mathematical model, we replicated a spirometry test by modifying the controller output (*R*_p_) to simulate a deep breath (see Supporting Information for details of the implementation). The obtained air inflow rate is given in Figure 6a. The time up to *t*_1_ correspond to the TPSS and a deep inhalation starts at *t*_1_. At *t*_2_ the air is exhaled out quickly and the expiratory flow rate is plotted against volume in Figure 6 (b, spirogram). The simulation was designed to match the FVC value of 4 L given in.^53^ The PEFR obtained is 6.7 L/s, and  which is close to the standard population-average.^53^ It may also be noted that the terminal part of the spirogram has a slope that is almost constant, another characteristic of healthy lung function.^54^

## 4 Model Adaptation to Represent a COPD Patient

Having verified the model to be qualitatively representative of the normal (healthy) cardiorespiratory system, the next goal is to adapt the model to represent a COPD patient. For this, we first identified the components of this model that were associated with the manifestations of COPD. In this section, we explain the procedure followed to identify and perform a quantitative analysis of the COPD-related components of the model.

### 4.1 Identification of COPD-Related Components in the Cardiorespiratory Model

COPD is a heterogeneous, multicomponent disease causing damage to different parts of the cardiorespiratory system. In this work, we hypothesize that the three manifestations of COPD namely, (1) airway obstruction, (2) emphysema, and (3) PH are caused due to a malfunction of the system, which can be represented as parametric adaptations in the systems biology model. Additional patient data is required to confirm/reject this hypothesis. In the following sections, we analyze the physiology of these manifestations, and identify the parameters associated with them. It may be noted that such parameters are often referred to as biomarkers or factors in biomedical research.^55^

#### 4.1.1 Airway Obstruction

Airflow in the lungs is the result of the balance between the elastic recoil of the lungs promoting flow and the resistance of the airways that limits flow. In COPD patients, and in particular people with *small airways disease*, excessive mucus (usually as a result of long-term smoking) narrows the bronchi. Consequently, the airway resistance increases, resulting in flow limitation. This COPD symptom can be replicated in simulation by increasing the value of the parameter representing “airway resistance” (*R* in eq S.3), from the “nominal” value for a healthy subject to a value high enough to change the process variables, *q* (air flow rate), sufficiently to produce effects matching what occurs in COPD patients.

#### 4.1.2 Lung Hyperinflation

Alveoli in the lungs are separated from one another by the alveolar septum. In *emphysema*, alveoli coalesce due to destruction of the septum, with the following two consequences. First, since the alveolar chambers in coalesced alveoli will now be much larger than the original distinct alveoli, the total surface area per volume of the respiratory membrane decreases. Second, due to the loss of elastic recoil, air sacs in the lungs lose their ability to function and exhale the air properly. As carbon dioxide is trapped in the lungs, fresh air flowing into the lungs pushes the walls of the lung further out with each new breath. This lack of air transfer causes the lungs to expand and lose their elasticity even further. The loss of elasticity causes more carbon dioxide to remain in the lungs leaving less space for fresh air and causing shortness of breath. Over time, the muscles and ribs surrounding the lungs are forced to stretch to fit the overexpanded lungs. The diaphragm, the major muscles used for breathing, becomes flattened and loses its ability to function properly. In the systems biology model, we denote the elasticity of air sacs in the lungs by the parameter *E*_T_ (total elastance) and the unloaded lung volume by *V*_0_. Changes in *E*_T_ and *V*_0_, result in substantial changes in physiological variables such as total lung capacity (TLC) and residual volume which are of interest in emphysema.

#### 4.1.3 Pulmonary Hypertension

PH^9^ is a type of high blood pressure that affects the pulmonary arteries and the right side of the heart. PH is a common complication in COPD wherein, pulmonary arterioles and capillaries become narrowed, blocked, or destroyed, making it harder for blood to flow through the lungs, thereby raising pressure within the lungs’ arteries. As the pressure builds, the heart’s right ventricle must work harder to pump blood through the lungs, eventually causing the heart muscles to weaken and ultimately fail. In the systems biology model, we identified the parameter *R*_pa_ (pulmonary artery resistance) as another COPD-related parameter, the increase of which causes the corresponding increase in the pulmonary artery pressure (*P*_pa_)—an indicator of PH.^56^

### 4.2 Quantitative Analysis on the COPD-Related Parameters

Having identified important parameters of the systems biology model that are linked to COPD manifestations, our next goal is to determine what changes to the associated parameters are required for the manifestation of COPD responses. We start by changing the value of a COPD-related parameter while keeping the other parameters constant. We monitor the changes in the COPD variables that are good indicators of the symptom associated with the varying parameter until we observe a COPD-like response in the variables. The parameter value at which COPD manifestation occurs is representative of the disease state. The application of this approach to each parameter is described in the following sections.

#### 4.2.1 Airway Resistance (*R*)

In this work, so far, the airway resistance (*R*) has been assumed to be a constant. Although in reality, the resistance has a dependence on the volume of the lung (channels become narrower as the lung volume reduces), this effect is minimal above the functional residual capacity (FRC) in healthy individuals.^57^ As the lung volume is usually above the FRC (black dotted line in Figure 3a), we could ignore the effect and assume the resistance to be a constant. On the other hand, in COPD patients, not only does the airway resistance increase, but the sensitivity of the airway resistance to lung volume also becomes more pronounced.^24^ To capture this behavior, the airway resistance in the model of a COPD patient was defined as a function of the lung volume as given by eq 1. Here, α is a parameter indicating the severity of the disease and at α = 0, the airway resistance becomes a constant (healthy individual). At positive values of α, the airway resistance has an inverse relationship with the alveolar volume, *V*_A_.
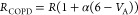
1

**Figure 7:**
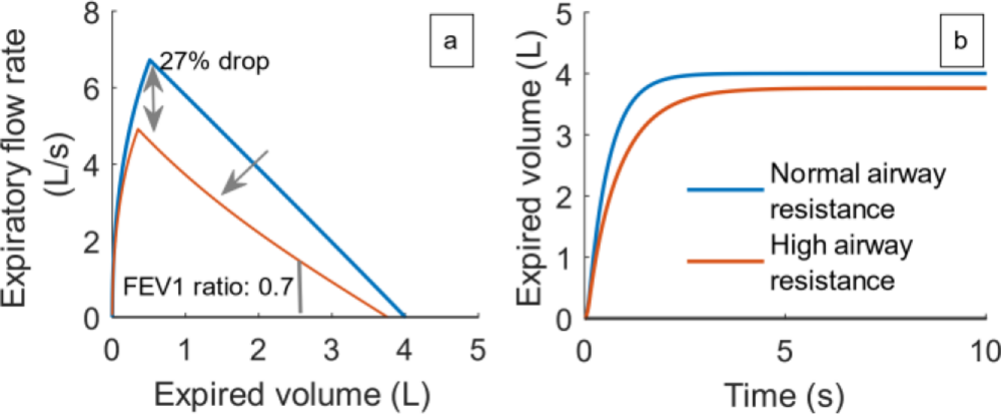
(a) Spirogram showing the air outflow rate (−*q*) as a function of the expired volume at normal (blue line) and higher (and variable, α = 0.205; red line) airway resistance. (b) Expired volume against time, in both healthy (blue) and disease (red) states.

To identify the α (and thereby the *R*_COPD_) value that is diagnostic of COPD, we used a diagnostic criterion that is widely used:^1^. The “spirometry test” (see Supporting Information for details) was repeated for different values of α until the FEV1 ratio decreased to 0.7. Note that, in the spirometry test, *V*_A_ has an inverse relation with the expired volume *V*_E_ during the exhalation. This follows the relation: *V*_A_ = TLC – *V*_E_. At α = 0.205 – corresponding to an 82% increase in airway resistance at a lung volume of 2*L* – the FEV1 ratio reduced to the desired level (Figure 7a). Due to the increase in airway resistance, the PEFR also dropped by about 27%, in accordance with alternative diagnostic criteria discussed in Jackson and Hubbard^14^ (20% decrease in PEFR). The terminal part of the spirogram also developed an inward curvature which is a hallmark of COPD.^58^Figure 7b gives the dynamics of expired volume obtained from the “spirometry test”. The total expired volume (FVC) is reduced in the case of high airway resistance. Though it has been hypothesized that the reduction in FVC could potentially be a biomarker in COPD, this is yet to be clinically validated.^59^

#### 4.2.2 Chest Elastance and Unloaded Lung Volume (*E*_T_, *V*_0_)

COPD patients with emphysema exhibit an increase in their lung volumes (*V*_A_) and a significant amount of work has been done on recording these changes, e.g., by Biselli et al.^60^ According to their results, TLC increases by about 14%, and the FRC increases by about 44% in the COPD state compared with the healthy state. We consider these volume changes as the threshold for the particular COPD manifestation.

**Figure 8:**
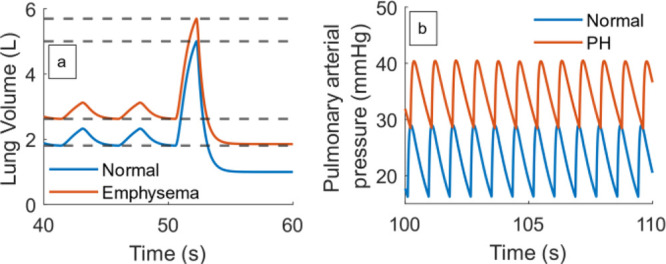
(a) Changes in lung volume (*V*_A_) corresponding to normal (blue) and emphysema (red). The two dashed lines on the top show the TLC recorded during a deep breath and those at the bottom show the FRC recorded during a normal breath. (b) Pulmonary arterial pressure in healthy (blue) and PH (red) states.

We simulated the model under the condition of breathing normal air for about 50.5 s to let the system reach TPSS. At *t* ≅ 50.5 s, a deep inhalation was simulated (see Supporting Information, Section S2). The FRC was identified as the lowermost point of the normal breathing cycles and the TLC was recorded at the end of inhalation where the lung volume is at its maximum capacity. The procedure was repeated for different values of *E*_T_ and *V*_0_ until an 820 mL increase in FRC and a 700 mL increase in the TLC were observed (Figure 8a). The solution converged to an unloaded lung volume (*V*_0, COPD_) of 1.06 L and a chest elastance (*E*_T, COPD_) of 2.75 mmHg/L.

#### 4.2.3 Pulmonary Artery Resistance (*R*_pa_)

In patients with COPD, PH is observed due to an increase in *R*_pa_. PH is defined to be the condition of *P*_pa_ > 35 mmHg.^56^ To determine the deviation in *R*_pa_ from normality necessary to precipitate PH, the model was simulated under the conditions of breathing normal air and a resting metabolic rate for 110 s. The average value of *P*_pa_ was recorded for the last 10 s. We repeat the simulation for different values of *R*_pa_ until an average *P*_pa_ of 35 mmHg was observed. This was observed at (originally 0.198). Figure 8b shows the dynamic response of *P*_pa_ to changes in *R*_pa_.

Table 2 summarizes the parameters chosen to capture the adaptations in COPD and their values in both healthy and disease states.

**Table 2:** Model Adaptation for COPD

condition	parameter affected	value: COPD	value: healthy	model eq
small airways disease	airway resistance [mmHg s /L]	*R*_COPD_ = 1 + 0.205(6 – *V*_A_)	*R* = 1	S.3
lung hyperinflation	unloaded lung volume [L] and chest elastance [mmHg/L]	*V*_0, COPD_ = 1.05	*V*_0_ = 0	S.4
		*E*_T, COPD_ = 2.75	*E*_T_ = 2.5	
PH	pulmonary resistance [mmHg.s/mL]	*R*_pa, COPD_ = 0.370	*R*_pa, COPD_ = 0.198	S.16

## 5 Discussion

The previous analysis is useful for identifying individual manifestations of COPD. However, COPD patients often exhibit a combination of symptoms, meaning changes in different parameters might coexist. To provide a comprehensive picture of the effects of COPD on the cardiorespiratory system, we need to develop a holistic approach which takes into account the effects of different parameters and their interactions together on the cardiorespiratory system. This objective can be achieved by proper design of simulation case studies, where the effects of parameter perturbations are studied individually and in combination.

We performed eight different simulations (details in the Supporting Information) corresponding to combinations of COPD symptoms. The response variables recorded were FVC, TLC, PEFR, FEV1 ratio, heart rate, *P*_pa_, respiratory rate, and minute ventilation. Among these variables, the first four are obtained by simulating deep breath and are referred to as respiratory characteristics. The last four are the stationary state values obtained from an extended period simulation, referred to as TPSS characteristics.

### 5.1 Respiratory Characteristics

**Figure 9:**
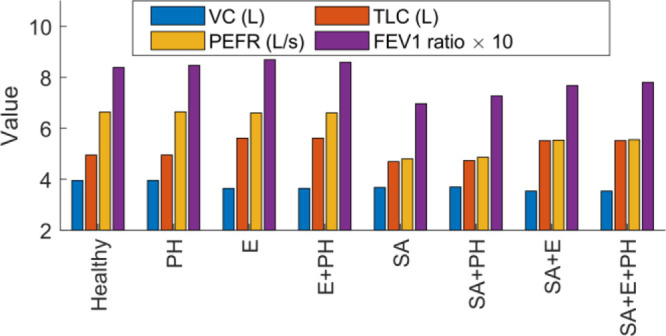
Respiratory characteristics—VC, TLC, PEFR, and FEV1 ratio in deep breath—under various manifestations of COPD. PH: Pulmonary hypertension, E: Emphysema, SA: Small airways.

The FVC, TLC, PEFR, and FEV1 ratios were identified for each case by simulating a deep breath (see the Supporting Information, spirometry test). The values observed under each condition are summarized in Figure 9. Changes in both airway resistance and lung elastance led to substantial changes in the vital capacity, while pulmonary arterial resistance had no noticeable effect on it. The TLC increased under emphysema and an increased resistance of the airways led to the decrease in both PEFR and the FEV1 ratio. On performing the t-tests, the airway resistance had a significant effect on all response variables and the lung elastance showed a significant effect on FVC, TLC and FEV1 ratio (see Table 3). Pulmonary arterial resistance did not have a significant effect on any of the responses considered because it affects the cardiovascular system primarily and not the respiratory characteristics.

**Table 3:**
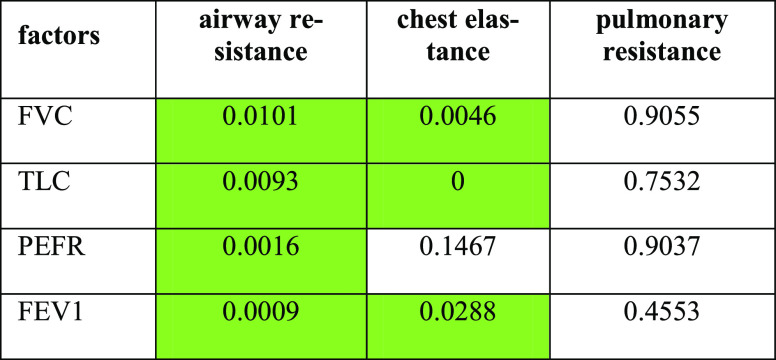
p-Values Associated with the Effect of Each Factor on the Respiratory Characteristics^a^

a Cells indicating a statistical significance (α = 0.1) are highlighted in green.

### 5.2 TPSS Characteristics

Next, the model was simulated for an extended period, under two different levels of inspired oxygen concentrations – 21 and 12%. Simulations were run for 1000 s under each condition and the reported TPSS values are the arithmetic mean of the final 100 s of these simulations. The objective of this study was to identify the disease characteristics that could adversely impact the patient under hypoxic environments such as high altitude or while on an airplane.

**Figure 10:**
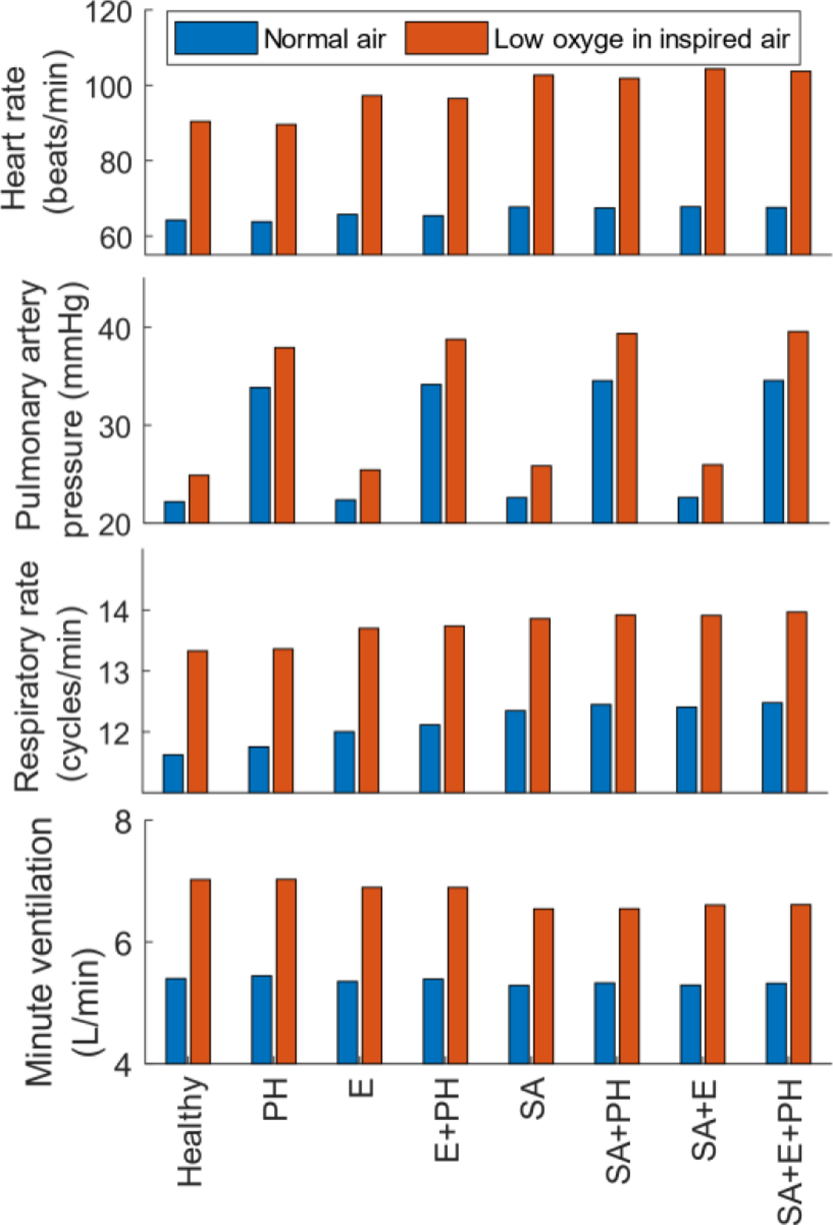
TPSS characteristics under various manifestations of COPD. PH: Pulmonary hypertension, E: Emphysema, SA: Small airways.

Figure 10 shows the heart rate, pulmonary artery pressure, respiratory rate and minute ventilation, both in normoxic environments (blue) and hypoxic environments (red). Under normoxic conditions, the changes in the airway resistance and the lung elastance lead to an increased heart rate, and all three factors contribute to an increased respiratory rate. Pulmonary resistance stands out as the major contributor to an increase in pulmonary arterial pressure under normoxic conditions. An increase in airway resistance has a major impact on the reduction in minute ventilation due to the difficulty in inhaling and exhaling air. On comparing the *healthy state* with the case of *increased pulmonary resistance* under normoxic conditions, there is a marginal decrease in heart rate (64.2–63.8 beats/min), and this is compensated for by a marginal increase in minute ventilation (5.40–5.45 L/s). Although the changes are minor, it is still interesting to note how the control systems transfer a part of the effort from the cardiovascular to the respiratory system in presence of a disturbance in the cardiovascular system (the change in pulmonary resistance).

In the models considered here, under hypoxic conditions, the TPSS characteristics remained at a higher level compared with those at normoxic conditions. To identify the COPD characteristics that have a significant impact on the *changes* in these response variables, t-tests were performed. Airway resistance was identified to have a significant impact on the changes in every response variable considered here (see Table 4). That is, when moving from a normoxic environment to a hypoxic environment, the changes observed in all four TPSS characteristics of a person with high airway resistance would be *significantly* different from that of a person with normal airway resistance. Likewise, the changes in heart rate and pulmonary pressure in a person with emphysema would be significantly different from that of someone with normal lung elastance. Pulmonary resistance has significant effects on the changes in pulmonary arterial pressure and respiratory rates.

**Table 4:**
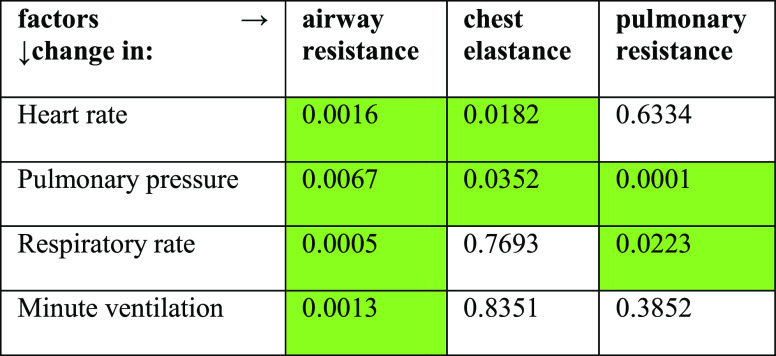
p-Values Associated with the Effect of Each Factor on the TPSS Characteristics^a^

a Green Highlight Indicating Statistical Significance (α = 0.1)

With all effects being significant, patients with high airway resistance appear to be at the highest risk from changes in atmospheric oxygen levels. Changes in pulmonary arterial pressures are affected by every factor considered here indicating that a decrease in the level of oxygen in air may induce higher stress on the cardiovascular system of all patients, irrespective of the expressed COPD manifestations.

## 6 Conclusions

Based on the premise that the regulation of physiological processes is achieved by biological control systems, we developed a control engineering framework for the modeling and analysis of COPD. Specifically we: (1) developed a representation of the human cardiorespiratory system in terms of control engineering components; (2) developed a computational dynamic model for each block with differential and algebraic equations representing known physiological functions; (3) verified the cardiorespiratory model with standard/literature data via simulations; (4) identified the model parameters associated with physiological characteristics of COPD and quantified the changes in them that are diagnostic of disease state; and (5) employed a statistical design of experiment approach to investigate the effects these parametric changes exert on the cardiorespiratory system under normal and hypoxic environments.

Our approach facilitated understanding underlying mechanisms of COPD, as it views COPD appropriately as a malfunction (or failure) of one or more components of the overall system. Among the COPD-related parameters studied here, an increase in airway resistance is identified to be the disease manifestation with maximum impact, affecting almost all response variables considered in this study. From the model simulations, it is also inferred that moving from normoxic to hypoxic environments induces a significantly higher stress on the cardiovascular system in COPD patients, irrespective of the manifestations observed in them.

The primary objective of the present work was to identify the qualitative differences in systemic response to different model adaptations (similar to^39^). In the future, we plan to add a few additional components in to the model such as the effect of changes in metabolic rates (see^61^ for preliminary results) and personalize the model for individual patients through identifying parameters using remote patient monitoring data.

## Supporting Information Available

Model equations, parameters, and the method for simulating the spirometry test and performing the multivariate analysis (PDF)
