# Supplementary material for: Systems Engineering Approach to Modeling and Analysis of Chronic Obstructive Pulmonary Disease
Source: ACS Omega. 2023 May 26;8(23):20524–35. doi: 10.1021/acsomega.3c00854 (PMC10268641; doi:10.1021/acsomega.3c00854)
Supplement: Supplementary file 1 — ao3c00854_si_001.pdf [file ao3c00854_si_001.pdf]

A Systems Engineering Approach to Modeling and Analysis of Chronic Obstructive Pulmonary Disease (COPD)

Varghese Kurian[1,‡], Navid Ghadipasha[1,‡,†], Michelle Gee[1,2], Anais Chalant[3], Teresa Hamill[3,†], Alphonse Okossi[3], Lucy Chen[3], Bin Yu[3, †], Babatunde A. Ogunnaike[1], Antony N. Beris[1,*]

[1]Department of Chemical and Biomolecular Engineering, University of Delaware, Newark, DE 19716, USA
[2]Daniel Baugh Institute of Functional Genomics/Computational Biology, Department of Pathology and Genomic Medicine, Thomas Jefferson University, Philadelphia, PA 19107, USA
[3]American Air Liquide Inc., Innovation Campus Delaware, Newark, DE 19702, USA

SUPPORTING INFORMATION

S1. Control engineering model of the cardio-respiratory system

Here we describe the model equations used to represent different physiological processes in the human cardio-respiratory system. (Main Text, Figures 1 & 2).

S1.1. Process: Lungs and Cardiovascular System Compartments

There are three main processes that occur in the lungs and heart to deliver oxygen to and eliminate carbon dioxide from the body cells. Ventilation, in which inhalation and exhalation occur, diffusion, which is the spontaneous movement of molecules between the gas in the alveoli and the bloodstream in the lung capillaries, and perfusion, the process by which the heart circulates blood throughout the cardiovascular system[1]. We describe the equations used for modeling each of these in the following paragraphs.

S1.1.1. Ventilation

It is assumed that the lung is a single compressible container which can support gas exchanges via blood flowing to and from the pulmonary capillaries. The flow of air in and out of the lungs (q) is driven by the difference between the mouth pressure (P_m) and the alveolar pressure (P_A). Among these, the mouth pressure remains constant, and the alveolar pressure varies with time, depending on the total inflow of gases into the lung (Q_A) and the pleural pressure (P_L). Figure S1 gives a schematic describing these processes and the mathematical relations are summarized by Equations S.1 – S.4. These equations are based on the works of Ben-Tal and others[2, 3].

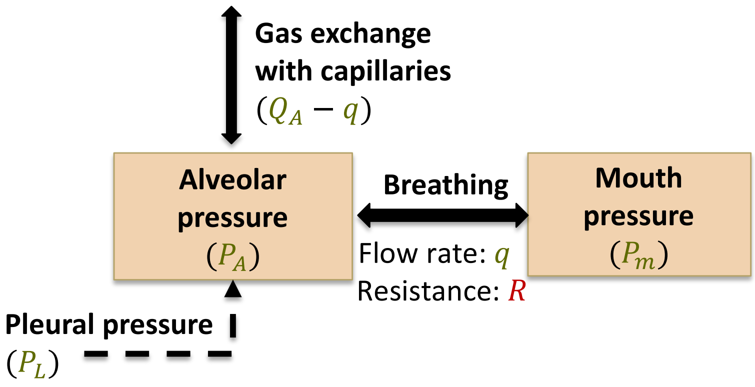

Figure S1 A schematic of the blocks involved in ventilation.

$$\frac{dP_A}{dt} = \frac{P_m E_T}{P_A} Q_A + \frac{dP_L}{dt} \tag{S.1}$$

$$Q_A = q + D_{CO2}(p_{CO2,Sa} - p_{CO2,al}) + D_{O2}(p_{O2,Sa} - p_{O2,al}) \tag{S.2}$$

$$q = \frac{P_m - P_A}{R} \tag{S.3}$$

$$V_A = \frac{P_A - P_L}{E_T} + V_0 \tag{S.4}$$

S1.1.2. Diffusion

In the alveoli, oxygen and carbon dioxide are exchanged between the alveolar air and pulmonary capillaries through diffusion as shown in Figure S2. Ben-Tal[2, 3] assumed the volume of the lung capillaries to be same as the stroke volume, allowing the blood to remain at the capillaries for the duration of a heartbeat while undergoing gas exchange. This assumption allowed them to reinitialize the differential equations at every heartbeat to account for blood circulation. In the present work, we modified their model, and integrated it with the model of the cardiovascular system[4] to account for the blood circulation in the pulmonary capillaries.

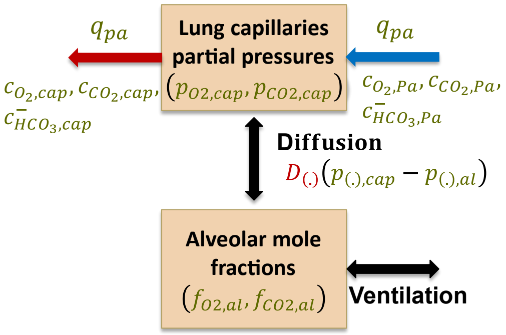

Figure S2 Schematic showing the key variables involved in the diffusion of gases between alveolar air and the lung capillaries.

The relations used to model the diffusion and the subsequent changes in gas concentrations are given by Equations S.5 – S.15. Equations S.5 and S.6 describe the change in mole fractions of oxygen $(f_{O2,al})$ and carbon dioxide $(f_{CO2,al})$ in the alveolar air due to the diffusion and ventilation. The alveolar partial pressures of gases $(p_{(.),al})$ are calculated from the mole fraction of gases and the alveolar pressure, after subtracting the partial pressure of water vapor p_w. The inspired volume V_i represents the amount of air inhaled in the current respiratory cycle and is brought to zero during exhalation as defined by Equation S.9. The mole fraction of oxygen in the

inspired air $(f_{O2,i})$ is assumed to be the same as the concentration of oxygen in the lung dead space in the early phase of inhalation. Later, after all air in the dead space is inhaled, it becomes equal to the mole fraction of atmospheric air. Finally, during exhalation, $f_{O2,i}$ is made equal to the mole fraction of alveolar oxygen (Equation S.10). Equations S.11 and S.14 model the changes in partial pressure of oxygen $(p_{O2,cap})$ and carbon dioxide $(p_{CO2,cap})$ in the lung capillaries due to diffusion and circulation of blood. The integration of the terms for circulation into these equations is based on the work by Ellwein et al[4]. The functions $\tilde{f}$ and g are the saturation functions for oxygen in blood[3, 4]. The last equation (Equation S.15) models the change in bicarbonate ions in the blood.

$$\frac{df_{O2,al}}{dt} = \frac{1}{V_A}\Big[D_{O2}\big(p_{O2,cap} - p_{O2,al}\big) + q\big(f_{O2,i} - f_{O2,al}\big) - f_{O2,al}\Big(D_{CO2}\big(p_{CO2,cap} - p_{CO2,al}\big) + D_{O2}\big(p_{O2,cap} - p_{O2,al}\big)\Big)\Big] \tag{S.5}$$

$$\frac{df_{CO2,al}}{dt} = \frac{1}{V_A}\big[D_{CO2}\big(p_{CO2,cap} - p_{CO2,al}\big) + q\big(f_{CO2,i} - f_{CO2,al}\big) - f_{CO2,al}(D_{O2}\big(p_{O2,cap} - p_{O2,al}\big) + D_{CO2}\big(p_{CO2,cap} - p_{CO2,al}\big))\big] \tag{S.6}$$

$$p_{O2,al} = f_{O2,al}(P_A - p_w) \tag{S.7}$$

$$p_{CO2,al} = f_{CO2,al}(P_A - p_w) \tag{S.8}$$

$$V_i(t) = \begin{cases} \dfrac{(2P_m - P_A(t) - P_A(t-\Delta t))}{2R}\Delta t + V_i(t-\Delta t) & if \quad (P_m - P_A) \geq 0 \\ 0 \qquad if \qquad (P_m - P_A) < 0 \end{cases} \tag{S.9}$$

$$f_{O2,i} = \begin{cases} f_{O2,m} & if \quad V_i > V_D \quad and \quad (P_m - P_A) > 0 \\ f_{O2,d} & if \quad V_i \leq V_D \quad and \quad (P_m - P_A) > 0 \\ f_{O2,al} & if \quad (P_m - P_A) \leq 0 \end{cases} \tag{S.10}$$

$$\frac{dp_{O2,cap}}{dt} = \frac{D_{O2}}{V_{cap}\,\sigma_{O2}}\left(1 + \frac{4T_h}{\sigma_{O2}}\frac{d\tilde{f}(p_{O2,Pa})}{dp_{O2,Pa}}\right)^{-1}\big(p_{O2,al} - p_{O2,cap}\big) + \frac{q_{pa}}{V_{cap}g\big(p_{O2,cap}\big)}c_{O2,cap} - \frac{q_{pa}}{V_{cap}g\big(p_{O2,Pa}\big)}c_{O2,Pa} \tag{S.11}$$

$$\tilde{f}\big(p_{O2,cap}\big) = \frac{LK_T\sigma p_{O2,cap}\big(1 + K_T\sigma p_{O2,cap}\big)^3 + K_R\sigma p_{O2,cap}\big(1 + K_R\sigma p_{O2,cap}\big)^3}{\Big(L\big(1 + K_T\sigma p_{O2,cap}\big)^4 + \big(1 + K_R\sigma p_{O2,cap}\big)^4\Big)} \tag{S.12}$$

$$\frac{dg\big(p_{O2,cap}\big)}{dp_{O2,cap}} = 2K_{O2}k_{O2}e^{-k_{O2}p_{O2,cap}}\big(1 - e^{-k_{O2}p_{O2,cap}}\big) \tag{S.13}$$

$$\frac{dp_{CO2,cap}}{dt} = \frac{D_{CO2}}{\sigma_{CO2}V_{cap}}\left(p_{CO2,al} - p_{CO2,cap}\right) + \frac{\delta l_2}{\sigma_{CO2}} hc_{HCO3,cap}^{-} - \delta r_2 p_{CO2,cap} + \frac{q_{pa}}{V_{cap}} p_{CO2,Pa} - \frac{q_{pa}}{V_{cap}} p_{CO2,cap} \tag{S.14}$$

$$\frac{dc_{HCO3,cap}^{-}}{dt} = \delta r_2 \sigma_{CO2} p_{CO2,cap} - \delta l_2 hc_{HCO3,cap}^{-} + \frac{q_{pa}}{V_{cap}}\left(c_{HCO3,cap}^{-} - c_{HCO3,Pa}^{-}\right) \tag{S.15}$$

S1.1.3. Circulation

As shown in Figure S3, the cardiovascular system is represented by a closed circuit with three arterial compartments, three venous compartments, and two ventricular compartments. These components are coupled with the lungs and the tissue compartments, which are responsible for the exchange of oxygen and carbon dioxide. Each cardiovascular compartment yields a representative transmural pressure $P(t)$, volume $V(t)$ and a constant compliance C. Compartments are separated by resistors R, analogous to an RC-circuit.

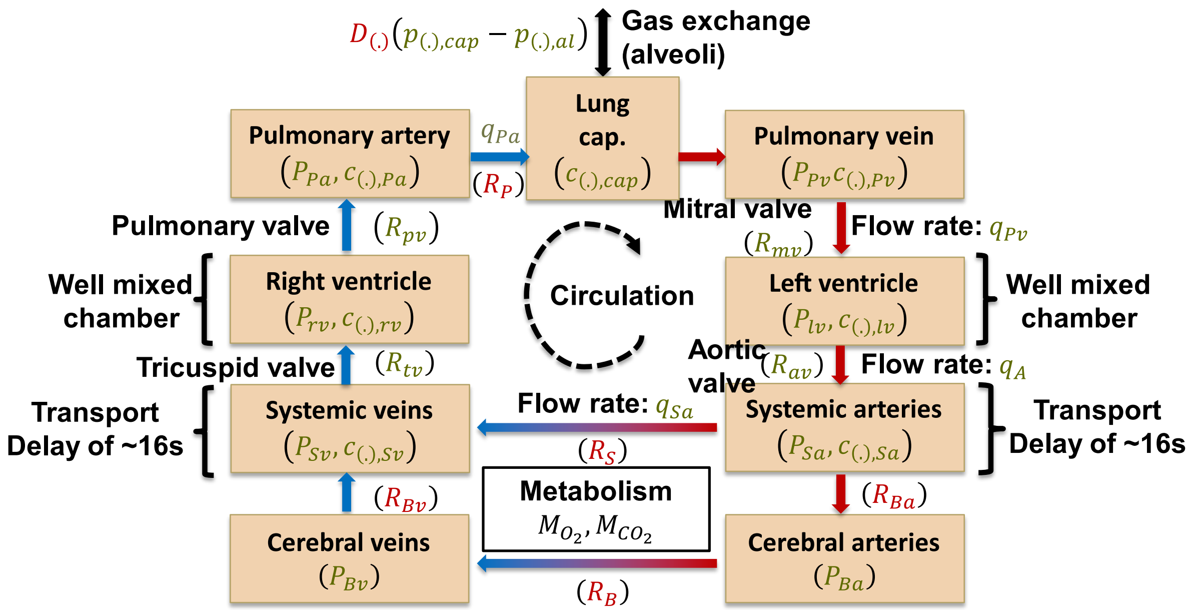

Figure S3 Schematic showing the key variables involved in the perfusion process. The R's denote the resitances, the q's denote the blood flow rate, and the c's denote the blood gas concentrations. The M's denote the metabolic rates (input variables). The resistances offered by the valves in the heart are functions of the pressures in adjacent compartments and therefore, vary with time.

As described by Ellwein et al[4]., in each compartment, the dynamics of blood pressure and flow can be explained by the mass and momentum balance. For example, Equation S.16 defines the resistive flow of blood between the compartments - the *pulmonary vein* and the *left ventricle* as a function of the pressures in the respective compartments and the resistance offered by the mitral valve. Similar equations define the flow rates between each pair of compartments in the circulatory system. Among the resistances, the arterial and venous resistances are parameters that remain constant for the entire duration of a heartbeat. However, the resistances at the four valves of the heart (mitral, aortic, tricuspid, and pulmonary) have dynamics that mimic the opening and closing of valves. Equation S.17 describes the variation in resistance across the mitral valve depending on the pressures in pulmonary vein and left ventricle. The equations describing the other three valves are the same, except for the parameter values which are given in Section S3. The variation in pressure in each compartment is a function of the net blood flow into the compartment and the compliance associated with the compartment, as given by Equation S.18. It is assumed that the compliance of the compartments remains constant in time and the values used in this work are available in Section S4. The time varying profile of the ventricular elastances drive the flow of blood in the RC circuit through changes in the ventricular pressures, as defined by Equation S.19. Here P_{lv} is the left ventricular pressure, E_{lv} is the time varying elastance, V_{lv} is the ventricular volume, and $V_{lv,d}$ is the dead volume of the respective ventricle (similarly for the right ventricle).

$$q_{pv} = \frac{P_{pv} - P_{lv}}{R_{mv}} \quad \text{(S.16)}$$

$$R_{mv} = min\left[R_{mv,o} + e^{-2(p_{Pv} - p_{lv})}, R_{mv,c}\right] \quad \text{(S.17)}$$

$$\frac{dP_{Pv}}{dt} = (q_{Pa} - q_{Pv})/C_{Pv} \quad \text{(S.18)}$$

$$P_{lv}(t) = E_{lv}(t)\left[V_{lv}(t) - V_{lv,d}\right] \quad \text{(S.19)}$$

The variations in concentrations of blood gases in the compartments of the circulatory system is defined using standard material balance equations. Equations S.11, S.14 (lung capillaries), and S.20, S.21 (tissues) represent the exchange of blood gases between the circulatory system and the rest of the human body. In all other compartments, the difference between the convective transport in and out of the compartment equals the accumulation of gases inside the compartment. The left and right ventricles are represented as well mixed chambers of varying volume as defined by Equation S.22.

$$q_{sa}\left(c_{CO2,Sa} - c_{CO2,sv}\right) = \left(M_{S,CO2}\right) \tag{S.20}$$

$$q_{sa}\left(c_{O2,Sa} - c_{O2,Sv}\right) = \left(M_{S,O2}\right) \tag{S.21}$$

$$\frac{dc_{(.),lv}}{dt} = \frac{q_{Pv}\left(c_{(.),Pv} - c_{(.),A}\right)}{V_{lv}} \tag{S.22}$$

Due to the time required for circulating the blood in the human system, there is a delay in the time necessary for the oxygen rich blood from the lung to reach the tissues. A similar delay exists for carbon dioxide rich blood to travel from tissues to the lung. In the mathematical model, these are incorporated as variable transport delays in two compartments - systemic arteries and systemic veins. The transport delay (t_d) at time t in the systemic arteries is calculated by solving Equation S.23 (see [5]), where $\frac{1500}{q_A}$ is the *instantaneous delay* corresponding to the flow rate at time t. This gives an average delay ~16 s at resting metabolic rate, which is close to the values reported in [6]. Similarly for the systemic veins. There is zero delay in other compartments.

$$\int_{t-t_d(t)}^{t} \frac{q_A(\tau)}{1500} d\tau = 1 \tag{S.23}$$

S1.2. Sensor: Chemoreceptors

The afferent input into the respiratory control system is provided primarily by two groups of neural receptors: 1) peripheral arterial chemoreceptors; 2) central (brainstem) chemoreceptors[7]. The peripheral arterial chemoreceptors consist of the carotid and aortic bodies. The carotid bodies respond to the partial pressures of oxygen and carbon dioxide and the hydrogen ion concentration in the blood. The intensity of the response varies according to the severity of the arterial hypoxemia or acidosis. The central chemoreceptors respond primarily to alterations of hydrogen ion concentration in the cerebrospinal fluid (CSF) and medullary interstitial fluid. The contribution of these central chemoreceptors to ventilation depends on the factors that alter hydrogen flux in their vicinity, causing changes in their intracellular pH.

In the model, the process of sensing blood gas level and blood pH is represented by three linear static relationships (Equations S.24 – S.26). The first two equations show the relationship between the blood partial pressure of oxygen and carbon dioxide in the systemic arteries and in the brain, and their corresponding measurements ($p_{O_2,s}, p_{CO_2,s}$). The third equation relates the concentration of bicarbonate (as an indicator of blood pH) to the sensor output. An additive relation between the measurements made by the peripheral and central chemoreceptors are used, as suggested by Batzel et al[8]. The constant of proportionality, $K_{(.),s}$, is the sensor gain, which is assumed to be 1 for a healthy person.

$$p_{O_2,s} = K_{O_2,s} \frac{(p_{o2,sa} + p_{o2,B})}{2} \tag{S.24}$$

$$p_{CO_2,s} = K_{CO_2,s} \frac{(p_{Co2,sa} + p_{Co2,B})}{2} \tag{S.25}$$

$$c^{-}_{HCO3,s} = K_{HCO3^-,s} \frac{(c^{-}_{HCO3,sa} + c^{-}_{HCO3,B})}{2} \tag{S.26}$$

S1.3. Controller: Respiratory Control Center

Breathing rhythm in mammals is controlled by a neural central pattern generator (CPG) that consists of multiple groups of neurons in the pons and medulla[9]. These groups of neurons are classified according to their behavior during the different phases of the breathing cycle; i.e., early inspiration, inspiration, and expiration. There is an extensive amount of work on CPG models which tend to focus on the mechanisms of individual neurons during the three phases of breathing cycle[3, 10]. These models are based on the theory by Hodgkin-Huxley who developed the first quantitative model of the initiation and propagation of an electrical signal along a giant squid axon.

Here we used the model by Ben-Tal[3] for capturing the response of the respiratory control center. The mechanisms behind the generation of the controller response, $R_p(t)$, from the controller input, $p_{o_2,s}$ and $p_{co_2,s}$, can be explained by the two interacting components: (1) an oscillator which receives the signals from the respiratory sensors, $p_{o_2,s}$ and $p_{co_2,s}$, and generates the average spike rate (A) in the neurons (Equations S.27 – S.34); (2) an inspiratory pattern generator that receives the signal from the oscillator and transforms it into a ramp pattern of neural activity - $R_p(t)$ (Equations S.35 – S.36).

In the model of the oscillator, $\tilde{g}_t$ is a parameter affecting the conductivity of the sodium ions based on the changes in the blood gas concentrations and K_{ctrl} is a parameter of the inspiratory pattern generator responsible for the respiratory amplitude. Ben-Tal[3] tested Proportional-Integral (PI) functions to relate $\tilde{g}_t$ and K_{ctrl} to the respiratory sensors' input. The exact feedback mechanism in the physiological system is not known yet, but $\tilde{g}_t$ and K_{ctrl} can be calculated from the input-output data available on respiratory control. That is, the response of the respiratory controller (respiratory rate and minute ventilation) to different values of $p_{CO_2,Sa}$ and $p_{O_2,Sa}$ are available and these can be used to tune the controller parameters $\tilde{g}_t$ and K_{ctrl}. Plots showing the

variations in $\tilde{g}_t$ and K_{ctrl}, and alveolar ventilation in response to variations in blood gas levels are provided in Section S6. These are based on the data given by Guyton and Hall[11].

$$\frac{dA}{dt} = \alpha(1 - A) - \beta A + \gamma \quad \text{(S.27)}$$

$$\alpha = \tilde{g}_{nap}\overline{m}_p h_p \quad \text{(S.28)}$$

$$\beta = \tilde{g}_t + \tilde{g}_L \quad \text{(S.29)}$$

$$\gamma = \tilde{b}\tilde{g}_t + \tilde{E}_L\tilde{g}_L \quad \text{(S.30)}$$

$$\overline{m}_p = \frac{1}{1 + e^{(A-\tilde{\theta}_{mp})/\tilde{\sigma}_{mp}}} \quad \text{(S.31)}$$

$$\frac{dh_p}{dt} = \alpha_{hp}(1 - h_p) - \beta_{hp}h_p \quad \text{(S.32)}$$

$$\alpha_{hp} = \frac{1}{2\overline{\tau}_{hp}} e^{-(A-\tilde{\theta}_{hp})/2\tilde{\sigma}_{hp}} \quad \text{(S.33)}$$

$$\beta_{hp} = \frac{1}{2\overline{\tau}_{hp}} e^{(A-\tilde{\theta}_{hp})/2\tilde{\sigma}_{hp}} \quad \text{(S.34)}$$

$$R_p(t) = \begin{cases} K_{ctrl} & if\ A(t) > T_{r1}\ and\ R_p(t - \Delta t) < T_{r4}, \\ I_p & if\ A(t) > T_{r1} \\ 0 & if\ A(t) \leq T_{r2} \\ R_p(t - \Delta t) & if\ R_p(t - \Delta t) > T_{r3}\ and\ \left|\frac{A(t) - A(t - \Delta t)}{\Delta t}\right| < \varepsilon \end{cases} \quad \text{(S.35)}$$

$$I_p = I_l * \big(A(t - \Delta t) + A(t)\big) * \frac{\Delta t}{2} + R_p(t - \Delta t) \quad \text{(S.36)}$$

S1.4. Actuator: Lung Muscles Compartment

The lung is contracted and expanded by two types of muscle: (1) the diaphragm (abdominal muscles) which can have an upward or downward movement to shorten or lengthen the chest cavity; and (2) the external intercostal muscles which can elevate or depress the ribs to increase and decrease the anteroposterior diameter of the chest cavity. The cells in the abdominal and intercostal muscles translate the electrical signal that comes from the controller into mechanical contraction and determines the pleural pressure in the lungs. Here the muscles are considered a spring which can be excited by the controller signal. The dynamics of muscle displacement are

explained by Equations S.37 and S.38[3]. The controller feedback is now connected to the process compartment through the pleural pressure (P_L) (see Equation S.1).

$$\frac{dx_m}{dt} = -k_1 x_m + k_2 R_p(t) \tag{S.37}$$

$$P_L = P_m - P_{L0} - k_p x_m \tag{S.38}$$

S1.5. Controller: Heart Rate Control

As the primary focus of our work is the simulation of the respiratory system, the heart rate was explicitly computed from the blood gas concentrations (Equation S.39) rather than using a detailed model as in the case of respiratory control. This equation was taken from the work of Milhorn et al.[12], who found the relation between the cerebral blood flow and blood partial pressure of oxygen and carbon dioxide. We expanded this equation further to account for the total cardiac output and the heart rate by adding two adjustable parameters, i.e., the ratio of cerebral blood flow to the total cardiac output (CBF_f) and the amount of blood which is pumped at each heartbeat (H_c). The heart rate is then used to calculate the heart period (T), the systolic time interval (T_M) and the diastolic relaxation time (T_R) (Equation S.40).

$$H = \frac{1}{CBF_f * H_c}\left(750 + W\left(h_1(P_{CO2,Sa})^5 + i_1(P_{CO2,Sa})^4 + j_1(P_{CO2,Sa})^3 + p_1(P_{CO2,Sa})^2 + q_1 P_{CO2,Sa} + r_1 + f(g - P_{O2,Sa})^s\right)\right) \tag{S.39}$$

$$\begin{aligned} T &= \frac{1}{H}\,[\mathrm{s}] \\ T_M &= T_{M,f}.T \\ T_R &= T_{R,f}.T \end{aligned} \tag{S.40}$$

S1.6. Actuator: Heart muscles

The time-varying elastance of the heart muscles in the left and right ventricles is given by Equation S.41. The systolic and early diastolic phases of the cycle are represented as scaled trigonometric functions. The elastance is assumed to remain constant in the rest of the diastole.

$$E(t) = \begin{cases} (E_s - E_D)\left[1 - \cos\left(\frac{\pi t}{T_M}\right)\right]/2 + E_D & 0 \le t \le T_M \\ (E_s - E_D)\left[\cos\left(\frac{\pi(t - T_M)}{T_R}\right) + 1\right]/2 + E_D & T_M \le t \le T_M + T_R \\ E_D & T_M + T_R \le t \le T \end{cases} \quad \text{(S.41)}$$

S1.7. Actuator: Vasodilation

When a higher flow rate of blood is required in the human body, the blood vessels dilate to reduce the net resistance of the circulatory system and this phenomenon is termed vasodilation. To represent this, in the mathematical model, the resistance of the arteries was divided by factor ($1 \le \tau \le 2.3$) depending on the heart rate as given by Equation S.42. The relation was developed by fitting a sigmoidal function on existing data[13].

$$\tau = \frac{1 + 2.3\, e^{\frac{HR-120}{2.3}}}{1 + e^{\frac{HR-120}{2.3}}} \quad \text{(S.42)}$$

S2. Replicating a spirometry test

In this section, we describe the steps followed in replicating a spirometry test in the mathematical model of a healthy individual.

We replicated a spirometry test by manually manipulating the controller's ramp output, R_p (Equation S.37). R_p – the output of the controller – is an electrical neural signal that determines the contraction of lung muscle. When R_p is greater than 0 (R_p> 0) inhalation occurs and R_p equals 0 ($R_p = 0$) corresponds to exhalation during the natural breathing rhythm. We started by simulating the model under the condition of breathing normal air and resting metabolic rate. The simulation continued for almost 50.5 seconds until we reached a stationary state. At $t \cong 50.5\ s$, around the end of expiration in a respiratory cycle, a step change in the R_p signal was applied,

increasing it by a value of six (Figure S4 a). Higher R_p values resulted in a higher muscle displacement (x_m), lower pleural pressure (P_L), and thereby, deeper inhalation (Figure S4 b & c). Following the deep inhalation at $t \cong 52.5s$, we quickly reduced the R_p signal to a value of -20 for a short period of time. The purpose of this step was to rapidly bring down the muscle displacement – the model equivalent of the quick release of air at the end of a deep inhalation. Once the muscle displacement reached zero, R_p was increased to a higher (but negative) value of -1.6 and remained there until the end of the simulation. When R_p remains below zero, muscle displacement also turns negative, and a forced exhalation occurs. The specific changes required R_p to simulate the deep breath – the increase by 6 units during the inhalation, the decrease to -1.6 during the exhalation – were identified by solving for the perturbations that result in an FVC of 4 L (TLC of 5L and RV of 1L, Figure S4)[14].

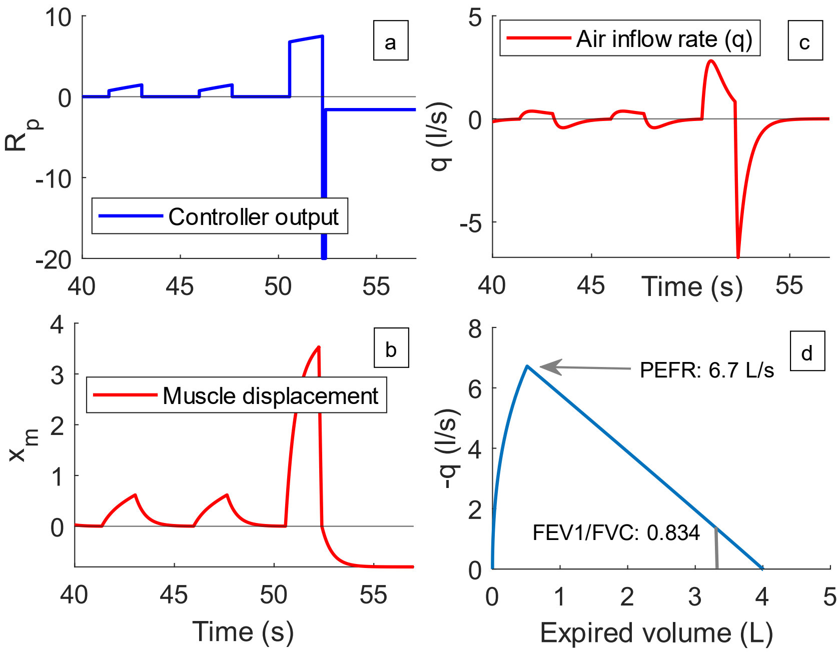

Figure S4 (a) Changes in R_p leading to changes in (b) muscle displacement and (c) air inflow rate. The spirogram is given in (d).

S3. Multivariable analysis of adaptations in COPD

This section describes the design of simulation case studies for performing a multivariable analysis of the different model adaptation in COPD: The steps involved are as follows:

1. We have three factors i.e. (i) airway resistance, (ii) chest elastance, and (iii) the resistance of the pulmonary arteries - that we would want to investigate the effects of, based on the response of the model.
2. Each factor has two levels, one corresponding to a healthy person and another representing a COPD patient.
3. Accounting for all combinations of the different factors, we have eight sets of parameters.
4. For each set of parameters, a deep breath was simulated to identify the spirometric variables.
5. In the next step, for each set of parameters, the model was simulated for (i) 1000 seconds under the condition of breathing normal air concentration and resting metabolic rate and (ii) 1000 seconds under an inspired oxygen concentration of 12%.
6. The values of the eight response variables that are good indicators of the COPD symptoms were recorded for each case. They are forced vital capacity (FVC), total lung capacity (TLC), PEFR (Peak Expiratory Flow Rate), $FEV1$ ratio, heart rate, pulmonary artery pressure (P_{pa}), respiratory rate, and minute ventilation.
7. Standardized t-tests were performed to identify whether the null hypothesis that the effect of each factor on the variable is zero can be rejected.

S4. Parameters and variables

This section lists all the parameters and variables that appear in this paper. They are listed in separate tables for each engineering module explained in the model development section. As the

parameter values are taken from multiple sources, the model response is indicative of the population average.

Table S 1 Parameters and variables - Lung compartment and alveolar air

Symbol	Definition	Type	Value [Units]	Ref
P_A	Alveolar pressure	Variable	[mmHg]	
V_A	Alveolar volume	Variable	[L]	
V_i	Inspired volume of the air	Variable	[L]	
V_D	Volume dead space	Parameter	0.15 [L]	[2]
Q_A	Net flux of air to the alveoli	Variable	[L/s]	
$f_{O2,al}$	Molar fraction of O2 in the alveoli	Variable	[-]	
$f_{O2,d}$	Molar fraction of O2 in the volume dead space	Variable	[-]	
$f_{CO2,al}$	Molar fraction of CO2 in the alveoli	Variable	[-]	
$f_{CO2,d}$	Molar fraction of CO2 in the volume dead space	Variable	[-]	
$f_{O2,i}$	Molar fraction of O2 in the inspired air	Variable	[-]	
$f_{CO2,i}$	Molar fraction of CO2 in the inspired air	Variable	[-]	
$p_{O2,al}$	Alveolar partial pressure of O2	Variable	[mmHg]	
$p_{CO2,al}$	Alveolar partial pressure of CO2	Variable	[mmHg]	
$c_{O2,cap}$	O2 Concentration in the lung capillaries	Variable	[mg_{gas}/mg_{blood}]	
$p_{CO2,cap}$	CO2 Partial pressure in the lung capillaries	Variable	[mmHg]	
$c_{\bar{H}CO3,cap}$	Carbonic acid concentration in the lung capillaries	Variable	[mol/L]	

$c_{O2,Pa}$	O2 Concentration in the pulmonary arteries	Variable	$[mg_{gas}/mg_{blood}]$	-
$p_{CO2,Pa}$	CO2 Partial pressure in the pulmonary artery	Variable	[mmHg]	-
$c_{\bar{H}CO3,Pa}$	Carbonic acid concentration in the pulmonary artery	Variable	[mol/L]	-
q	Air flow through the airways	Variable	[L/s]	
σ_{O2}	(Solubility of O2 in plasma)	Parameter	1.4×10^{-6}[mol/l.mmHg]	2
σ_{CO2}	(Solubility of CO2 in plasma)	Parameter	3.3×10^{-5}[mol/l.mmHg]	2
r_2	(Hydration reaction rate)	Parameter	0.12 [1/s]	2
l_2	(Dehydration reaction rate)	Parameter	164×10^3 [L/mol . s]	2
T_h	(Threshold hemoglobin concentration)	Parameter	2×10^{-3} [mol/L]	2
δ	(Acceleration rate)	Parameter	5000 [-]	15
h	(concentration of H^+)	Parameter	$10^{-7.4}$ [mol/L]	2
R	(Airways resistance to flow)	Parameter	1 [mmHg. s /L]	2
p_w	(Vapor pressure of water)	Parameter	47 [mmHg]	2
D_{O2}	(Diffusion capacity O2)	Parameter	3.5×10^{-4} [L/s . mmHg]	3
D_{CO2}	(Diffusion capacity CO2)	Parameter	7.08×10^{-3} [L/s . mmHg]	3
V_{cap}	(Capillaries volume)	Parameter	0.07 [L]	2
E_T	(Lung elastance)	Parameter	2.5 [mmHg/L]	2
V_0	Volume of the lungs when it is unloaded	Parameter	0 [L]	3 (see Sec 3.2)
K_T	Equilibrium constant in the saturation function of hemoglobin	Parameter	1.0×10^4 [L/mol]	2
K_R	Equilibrium constant in the saturation function of hemoglobin	Parameter	3.6×10^6 [L/mol]	2

L	Equilibrium constant in the saturation function of hemoglobin	Parameter	171.2×10^6 [-]	[2]

Table S 2 Parameters and variables - respiratory sensors

Symbol	Definition	Type	Value [Units]	Ref
$p_{o2,s}$	Blood partial pressure of O2 in the systemic arteries measured by arterial chemoreceptors	Variable	-	
$p_{co2,s}$	Blood partial pressure of CO2 in the Arteries Measured by arterial chemoreceptors	Variable	-	
$c^{-}_{HCO3,s}$	Concentration of Carbonic Acid (Representation of PH) measured by arterial chemoreceptors	Variable	-	
$K_{O_2,s}$	Sensor gain for oxygen	Parameter	1 (healthy person)	[16]
$K_{CO_2,s}$	Sensor gain for carbon dioxide	Parameter	1 (healthy person)	[16]
$K_{HCO3^-,s}$	Sensor gain for carbonic acid	Parameter	1 (healthy person)	[16]

Table S 3 Parameters and variables - respiratory control center

Symbol	Definition	Type	Value [Units]	Ref
A	Average measure of spike rate in the neurons population	Variable		
h_p	Inactivation gating of persistent sodium current	Variable		
m_p	Activation gating of persistent sodium current	Variable		

$R_p(t)$	Phrenic activity signal causing the lung muscle force	Variable		
$\tilde{g}_t$	Controller parameter responsible for respiratory frequency	Variable		Tuned using data in [11] (see Section S6)
K_{ctrl}	Controller parameter responsible for respiratory amplitude	Variable		Tuned using data in [11] (see Section S6)
$\tilde{g}_{nap}$	(Activating rate constant of A)	Parameter	133.33 [1/s]	[3]
$\tilde{g}_L$	(Inactivating rate constant of A)	Parameter	98 [1/s]	[3]
$\tilde{\theta}_{mp}$	(Parameter affecting $\bar{m}_p$)	Parameter	0.367 [-]	[3]
$\tilde{\sigma}_{mp}$	(Parameter affecting $\bar{m}_p$)	Parameter	-0.033 [-]	[3]
$\tilde{\theta}_{hp}$	(parameter affecting h_p)	Parameter	0.313 [-]	[3]
$\tilde{\sigma}_{hp}$	(parameter affecting h_p)	Parameter	0.04 [-]	[3]
$\bar{\tau}_{hp}$	(parameter affecting h_p)	Parameter	10 [s]	[3]
$\tilde{E}_L$	(parameter affecting the external drive of A)	Parameter	0.212 [-]	[3]
Tr_1	Threshold value in the ramp generation function	Parameter	0.35 [-]	[3]
Tr_2	Threshold value in the ramp generation function	Parameter	0.35 [-]	[3]
Tr_3	Threshold value in the ramp generation function	Parameter	0.3 [-]	[3]
Tr_4	Threshold value in the ramp generation function	Parameter	0.001 [-]	[3]

Table S 4 Parameters and variables - lung muscles (actuator)

Symbol	Definition	Type	Value [Units]	Ref

P_L	Pleural pressure	Variable	[mmHg]	
x_m	Muscle displacement	Variable	[m]	
P_m	Mouth pressure	Parameter	760 [mmHg]	3
P_{L0}	Difference between atmospheric and pleural pressure	Parameter	4.5 [mmHg]	2
k_1	Recoil rate constant of muscle	Parameter	2 [1/s]	3
k_2	Conversion constant	Parameter	1 [m/s]	3
k_p	Conversion constant	Parameter	2.5 [mmHg/m]	3

Table S 5 Parameters and variables - cardiovascular system

**Symbol**	**Definition**	**Type**	**Value [Units]**	**Ref**
q_{pv}	Pulmonary vein blood flow rate	Variable	[mL/s]	
P_{pv}	Pulmonary vein pressure	Variable	[mmHg]	
C_{pv}	Pulmonary venous compliance	Parameter	22.4 [mL/mmHg]	4
R_p	Pulmonary Resistance	Parameter	0.198 [mmHg.s/mL]	4
R_{mv}	Mitral valve Resistance	Variable	[mmHg.s/mL]	
$R_{\mathrm{mv},o}$	Open mitral valve resistance	Parameter	0.001 [mmHg.s/mL]	4
$R_{\mathrm{mv},c}$	Closed mitral valve resistance	Parameter	20 [mmHg.s/mL]	4
q_{lv}	Left ventricle blood flow rate	Variable	[mL/s]	
V_{lv}	Left ventricular volume	Variable	[mL]	
E_{lv}	Left ventricular elastance	Variable	[mmHg/mL]	
$E_{D,l}$	Left ventricular diastolic elastance	Parameter	0.0135 [mmHg/mL]	4
$E_{s,l}$	Left ventricular systolic elastance	Parameter	0.849 [mmHg/mL]	4

q_{sa}	Systemic artery<br><br>blood flow rate	Variable	[mL/s]	
P_{Sa}	Systemic arteries pressure	Variable	[mmHg]	
C_{sa}	Systemic arteries<br><br>Compliance	Parameter	2.4 [mL/mmHg]	[4]
R_s	Systemic Resistance	Parameter	1.08 [mmHg.s/mL]	[4]
R_{av}	Aortic Valve Resistance	Variable	[mmHg.s/mL]	
$R_{\mathrm{av},o}$	Open aortic valve valve resistance	Parameter	0.001 [mmHg.s/mL]	[4]
$R_{\mathrm{av},c}$	Closed aortic valve valve resistance	Parameter	20 [mmHg.s/mL]	[4]
$c_{CO_2,Sv}$	CO2 concentration in the systemic vein	Variable	[mg/mL]	
$c_{CO_2,\mathrm{Sa}}$	CO2 concentration in the systemic arteries	Variable	[mg/mL]	
$M_{S,CO2}$	Body CO2 tissue<br><br>metabolism	Variable	[mg/s]	
$M_{S,O2}$	Body O2 tissue<br><br>metabolism	Variable	[mg/s]	
q_{sv}	Systemic vein<br><br>blood flow rate	Variable	[mL/s]	
P_{Sv}	Systemic vein pressure	Variable	[mmHg]	
C_{sv}	Systemic venous Compliance	Parameter	39.9 [mL/mmHg]	[4]
R_s	Systemic Resistance	Parameter	1.08 [mmHg . s/mL]	[4]
R_{tv}	Tricuspid valve resistance	Variable	[mmHg . s/mL]	
$R_{\mathrm{tv},o}$	Open Tricuspid valve resistance	Parameter	0.001 [mmHg . s/mL]	[4]
$R_{\mathrm{tv},c}$	Closed Tricuspid valve resistance	Parameter	20 [mmHg . s/mL]	[4]
q_{rv}	Right ventricle<br><br>blood flow rate	Variable	[mL/s]	
V_{rv}	Right ventricular volume	Variable	[mL]	
E_{rv}	Right ventricular elastance	Variable	[mmHg/mL]	

$E_{D,r}$	Right ventricular diastolic elastance	Parameter	0.0667 [mmHg/mL]	[4]
$E_{s,r}$	Right ventricular systolic elastance	Parameter	3.38 [mmHg/mL]	[4]
q_{pa}	Pulmonary artery blood flow rate	Variable	[mL/s]	
P_{pa}	Pulmonary artery pressure	Variable	[mmHg]	
C_{pa}	Pulmonary arterial Compliance	Parameter	4.52 [mL/mmHg]	[4]
R_p	Pulmonary Resistance	Parameter	0.198 [mmHg.s/mL]	[4]
R_{pv}	Pulmonary valve Resistance	Variable	[mmHg.s/mL]	
$R_{\mathrm{pv},o}$	Open pv resistance	Parameter	0.001 [mmHg.s/mL]	[4]
$R_{\mathrm{pv},c}$	Closed pv resistance	Parameter	20 [mmHg.s/mL]	[4]
H	Mean Heart Rate	Variable	[beat/min]	
$T_{M,f}$	Fraction of time to end-systolic to the heart time period	Parameter	0.35	[4]
T_R, f	Fraction of ventricular relaxation time to the heart time period	Parameter	0.175	[4]
T	Heart Time Period	Variable	[min]	

Table S 6 Polynomial coefficients of Equation S.32 [12]

**Symbol**	**Value**
W	0.014
h_1	3.23E-6
i_1	-4.46E-4
j_1	2.25E-2
p_1	-4.79E-1
q_1	4.37
r	-43

f	-0.003
g	98
s	2.3

S5. Initial conditions

Initial conditions for the cardio-respiratory model. These are selected from the references (as indicated below) for the majority of the cases. In a few cases where the initial conditions were not readily available, these were chosen from the actual ranges of the variables.

Table S 7 Initial conditions

**Symbol**	**Meaning**	**Initial value**
P_A	Alveolar pressure	760 [mmHg][2]
$f_{O2,al}$	The molar fraction of O2 in the alveoli	0.13 [-][2]
$f_{CO2,al}$	The molar fraction of CO2 in the alveoli	0.055 [-] (0.053 used in [2])
$p_{O2,Sa}$	O2 partial pressure in the systemic arteries	90 [mmHg] (100 used in [4])
$p_{CO2,Sa}$	CO2 partial pressure in the systemic arteries	42 [mmHg] (40 used in [4])
$c_{\bar{H}CO3,Sa}$	bicarbonate concentration in the systemic arteries	0.028 [mol/L][2]
A	Average measure of spike rate in the neurons population	0.34 [-] (chosen from the final range in [3])
h_p	Inactivation gating of persistent sodium current	0.33 [-] (chosen from the final range in [3])
x_m	Muscle displacement	0 [-] (equilibrium value when no external drive)
P_{pv}	Pulmonary vein pressure	3.3 [mmHg][4]
P_{Sv}	Systemic vein pressure	6.6 [mmHg][4]

P_{Sa}	Systemic arteries pressure	79.5 [mmHg][4]
P_{pa}	Pulmonary artery pressure	20 [mmHg][4]
V_{lv}	Left ventricular volume	312 [mL][4]
V_{rv}	Right ventricular volume	100 [mL][4]

S6. Data used for defining controller parameters $\tilde{g}_t$ and K_{ctrl}

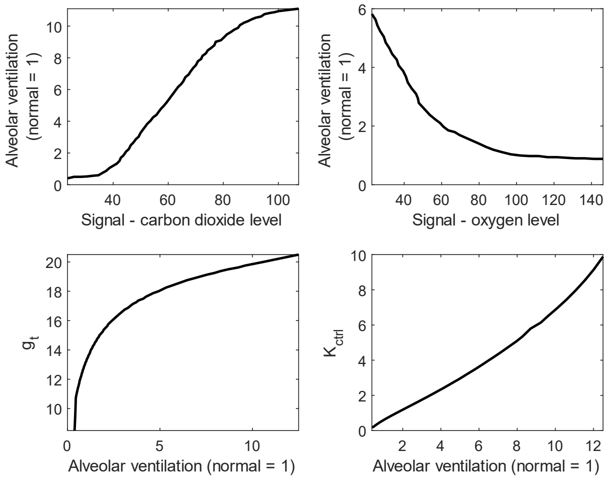

Figure S5 The data used for connecting the blood gas measurements to the controller parameters, $\tilde{g}_t$ and K_{ctrl} [11]

References

(1) Wagner, P. D. Pulmonary gas exchange. In *An Introductory Text To Bioengineering*, Chien, S., Chen, P. C., Fung, Y. C. Eds.; World Scientific, 2008.

(2) Ben-Tal, A. Simplified models for gas exchange in the human lungs. *J Theor Biol* **2006**, *238* (2), 474-495. DOI: 10.1016/j.jtbi.2005.06.005.

(3) Ben-Tal, A.; Smith, J. C. A model for control of breathing in mammals: coupling neural dynamics to peripheral gas exchange and transport. *J Theor Biol* **2008**, *251* (3), 480-497. DOI: 10.1016/j.jtbi.2007.12.018.

(4) Ellwein, L.; Pope, S.; Xie, A.; Batzel, J.; Kelley, C.; Olufsen, M. Patient-specific modeling of cardiovascular and respiratory dynamics during hypercapnia. *Mathematical biosciences* **2013**, *241* (1), 56-74.

(5) MATLAB. *Variable Transport Delay*. https://www.mathworks.com/help/simulink/slref/variabletransportdelay.html#f8-894946_sep_bqgafpc (accessed 2022 10/20).

(6) Kwon, Y.; Mariani, S.; Gadi, S. R.; Jacobs Jr, D. R.; Punjabi, N. M.; Reid, M. L.; Azarbarzin, A.; Wellman, A. D.; Redline, S. Characterization of lung-to-finger circulation time in sleep study assessment: the Multi-Ethnic Study of Atherosclerosis. *Physiological Measurement* **2020**, *41* (6), 065004.

(7) Caruana-Montaldo, B.; Gleeson, K.; Zwillich, C. W. The control of breathing in clinical practice. *Chest* **2000**, *117* (1), 205-225.

(8) Batzel, J. J.; Kappel, F.; Schneditz, D.; Tran, H. T. *Cardiovascular and respiratory systems: modeling, analysis, and control*; SIAM, 2007.
(9) Ramirez, J.-M.; Richter, D. W. The neuronal mechanisms of respiratory rhythm generation. *Current opinion in neurobiology* **1996**, *6* (6), 817-825. Richter, D. Neural regulation of respiration: rhythmogenesis and afferent control. In *Comprehensive human physiology*, Springer, 1996; pp 2079-2095.
(10) Butera Jr, R. J.; Rinzel, J.; Smith, J. C. Models of respiratory rhythm generation in the pre-Botzinger complex. I. Bursting pacemaker neurons. *Journal of neurophysiology* **1999**, *82* (1), 382-397. Rybak, I. A.; Paton, J. F.; Schwaber, J. S. Modeling neural mechanisms for genesis of respiratory rhythm and pattern. I. Models of respiratory neurons. *Journal of neurophysiology* **1997**, *77* (4), 1994-2006.
(11) Guyton, A.; Hall, J. Textbook of medical physiology, 11th. Elsevier Inc.: 2006.
(12) Milhorn Jr, H. T.; Benton, R.; Ross, R.; Guyton, A. C. A mathematical model of the human respiratory control system. *Biophysical Journal* **1965**, *5* (1), 27.
(13) Pawelczyk, J.; Hanel, B.; Pawelczyk, R.; Warberg, J.; Secher, N. Leg vasoconstriction during dynamic exercise with reduced cardiac output. *Journal of Applied Physiology* **1992**, *73* (5), 1838-1846. Park, J. H.; Gorky, J.; Ogunnaike, B.; Vadigepalli, R.; Schwaber, J. S. Investigating the effects of brainstem neuronal adaptation on cardiovascular homeostasis. *Frontiers in neuroscience* **2020**, *14*, 470.
(14) McKeever, T. M.; Weston, P. J.; Hubbard, R.; Fogarty, A. Lung function and glucose metabolism: an analysis of data from the Third National Health and Nutrition Examination Survey. *American journal of epidemiology* **2005**, *161* (6), 546-556.
(15) Holland, R.; Forster 2nd, R. Effect of temperature on rate of CO2 uptake by human red cell suspensions. *American Journal of Physiology-Legacy Content* **1975**, *228* (5), 1589-1596.
(16) Christie, C. R.; Achenie, L. E. K.; Ogunnaike, B. A. A control engineering model of calcium regulation. *The Journal of Clinical Endocrinology & Metabolism* **2014**, *99* (8), 2844-2853.
